# Mechanisms underlying patchy distribution pattern and spread of the invasive species *Solanum rostratum* (Solanaceae) in the Agro-Pastoral region of Northern China

**DOI:** 10.3389/fpls.2026.1809773

**Published:** 2026-05-13

**Authors:** Qian-Mei Wu, Rui Wang, Li-Fen Hao, Cheng-Dong Xu, Hui Wang, Ke-Jian Lin

**Affiliations:** 1 Key Laboratory of Biohazard Monitoring and Green Prevention and Control for Artificial Grassland, Ministry of Agriculture and Rural Affairs, and Inner Mongolia Key Laboratory of Grassland Protection Ecology, Grassland Research Institute, Chinese Academy of Agricultural Sciences, Hohhot, China; 2 State Key Laboratory for Biology of Plant Diseases and Insect Pests, Institute of Plant Protection, Chinese Academy of Agricultural Sciences, Beijing, China; 3 State Key Laboratory of Resources and Environmental Information System, Institute of Geographic Sciences and Natural Resources Research, Chinese Academy of Sciences, Beijing, China

**Keywords:** dispersal mechanisms, distribution pattern, invasive species, patchy distribution, *Solanum rostratum* Dunal

## Abstract

**Introduction:**

Understanding the mechanisms driving patchy distribution patterns is crucial for managing invasive species. This study investigates the multi-vector dispersal dynamics underlying the patchy spread of *Solanum rostratum* in the Agro-Pastoral Region of Northern China.

**Methods:**

We integrated spatial analysis of historical and field‑collected occurrence data, mechanistic simulations, and field experiments to quantify dispersal by wind, vehicles, and animals. A Gamma Generalized Linear Mixed Model (GLMM) was used to attribute variation in empirically derived minimum arrival speeds to different vectors and their interactions.

**Results:**

We identified 103 spatially clustered patches with strong directional alignment to roads but not rivers. Wind‑mediated dispersal was limited (<0.12 km/year), whereas vehicle‑ and animal‑mediated epizoochory showed higher potential (0.08–0.58 and 0.44–0.56 km/year, respectively). Statistical models attributed 64.3% of the variance in spread speed to these three vectors, with vehicle dispersal being the most influential single factor. Synergistic interactions among vectors significantly enhanced local, within‑patch spread. However, observed long‑distance, inter‑patch dispersal speeds (up to 1,746 km/year) vastly exceeded the capacity of any natural vector or their synergy, implicating human‑mediated transport of contaminated materials as the primary driver of regional colonization.

**Discussion:**

These findings demonstrate that patchy invasions can arise from distinct mechanisms operating at different scales: local synergistic dispersal among natural and anthropogenic vectors, and long‑distance jump dispersal via human activities. Effective management therefore requires dual strategies targeting local vector synergy and regional pathways of human‑assisted spread.

## 1 Introduction

Invasive species represent one of the most significant global environmental challenges of the 21st century, with profound economic, ecological, and human health impacts (Pimentel et al., 2000; MacIsaac et al., 2011). These non-native species disrupt ecosystems, outcompete native biodiversity, and cause substantial agricultural losses (Pimentel et al., 2005; Baker, 2016). Globalization and expanding trade networks have amplified their spread, increasing the likelihood of introductions into new environments (Anderson et al., 2015; Early et al., 2016; Sardain et al., 2019). Once established, invasive populations often reach ecological thresholds that render control efforts ineffective, underscoring the urgency of early intervention (Choi, 2012).

The early invasion stages—introduction, colonization, and spread—are critical for management, yet detection during these phases remains challenging due to small, dispersed populations (Early et al., 2016). Understanding spatiotemporal dynamics, including distribution patterns and dispersal mechanisms, is therefore essential for identifying high-risk areas and designing targeted strategies (Choi et al., 2017; Petrovskaya and Zhang, 2020). Historically, invasive spread was conceptualized as a continuous advancing front, as modeled by Kolmogorov et al. (1937) and Skellam (1951). However, contemporary studies reveal discontinuous, patchy invasion edges characterized by isolated populations of varying densities—a pattern that complicates traditional control approaches (Williams and Krock, 2012; Wu et al., 2022).

Patchy distributions arise from two complementary mechanisms. The first is jump dispersal, which involves long-distance movement (e.g., human-mediated transport, wind, or animal vectors) that establishes isolated satellite populations. The second is local dispersal, driven by short-distance expansion of existing patches via propagule movement (e.g., seeds, larvae) (Rodrigues et al., 2015). The interplay of these processes generates dynamic, heterogeneous distributions that resist containment and accelerate ecosystem colonization (Nathan et al., 2008; Horvitz et al., 2014). Critically, synergistic interactions among vectors can amplify invasion rates, as demonstrated for some alien species (Nathan, 2006; Wang et al., 2011), yet their relative contributions remain poorly quantified.

Two unresolved questions hinder management: (1) How can we identify effective dispersal vectors for specific invasive species in particular regions? and (2) What are the relative contributions of these vectors to spread dynamics? While past research has focused on single-vector assessments (e.g., wind-driven seed dispersal), such approaches oversimplify natural systems where multiple vectors interact. For instance, *Ageratina adenophora*’s rapid invasion is driven by combined wind, water, and human activity—a complexity overlooked in single-vector studies (Wang et al., 2011). This gap limits our ability to predict spread rates or prioritize high-impact vectors for control.

Emerging hybrid frameworks integrate geostatistical analyses, empirical data (e.g., annual occurrence tracking), and mechanistic models (e.g., wind-, water-, and human-mediated dispersal simulations) to resolve multi-vector dynamics (Horvitz et al., 2014, 2017). However, these methods often require large datasets, posing logistical challenges. Here, we adopt such an integrative approach to investigate *Solanum rostratum* Dunal, a highly invasive North American plant that has colonized ten northern Chinese provinces since its 1981 introduction. The invasion success of *S. rostratum* is driven by a combination of dispersal vectors, including wind, water, animal-mediated transport, and human activities (e.g., vehicular movement and agricultural trade), which facilitate both localized patch expansion and long-distance dispersal events (Rushing et al., 1985; Jackline and Matzrafi, 2021). Preliminary surveys and an examination of early distribution records of *S. rostratum* in northern China indicated a recurring spatial association of its populations with road networks—a pattern commonly observed for invasive species with adhesive propagules that exploit human transportation corridors (Christen and Matlack, 2009). Coupled with its known seed morphology (barbed spines) that is highly conducive to external attachment (Eminniyaz et al., 2013), these observations led us to formulate the *a priori* hypothesis that human-mediated dispersal, particularly via vehicles, is a critical yet poorly quantified vector facilitating its rapid spread and patch formation in the region. We hypothesize that synergistic vector interactions drive the species’ rapid spread and heterogeneous spatial patterns.

This study addresses three objectives: (1) to map the spatiotemporal distribution of *S. rostratum* and quantify patch formation and expansion trends; (2) to assess seed dispersal distances mediated by potential vectors such as wind, animals, and vehicles for intra- and inter-patch spread; and (3) to compare observed invasion front speeds with single- or multiple-vector dispersal speeds to identify primary drivers of rapid expansion and evaluate the relative contributions of dispersal vectors (e.g., wind, animals, vehicles) to intra- and inter-patch dynamics. By resolving these mechanisms, we aim to advance ecological understanding of multi-vector dispersal dynamics and inform strategies to mitigate *S. rostratum*’s impacts on agricultural and ecological security in northern China.

## 2 Materials and methods

To address the three research objectives, we designed an integrated study combining spatial analysis, field and simulation experiments, and statistical modeling. The workflow proceeded in three sequential phases. First, to map spatiotemporal distribution patterns and delineate invasion patches (Objective 1), we compiled and analyzed historical and contemporary occurrence data using geospatial techniques. Second, to quantify the dispersal potential of wind, vehicle, and animal vectors (Objective 2), we conducted controlled adhesion experiments, field measurements, and mechanistic simulations for each pathway. Third, to identify the dominant drivers of spread by comparing observed speeds with vector capacities (Objective 3), we calculated minimum arrival speeds from the occurrence data and employed generalized linear mixed models to partition the variance explained by different vectors and their interactions.

### 2.1 Study area

**Figure 1:**
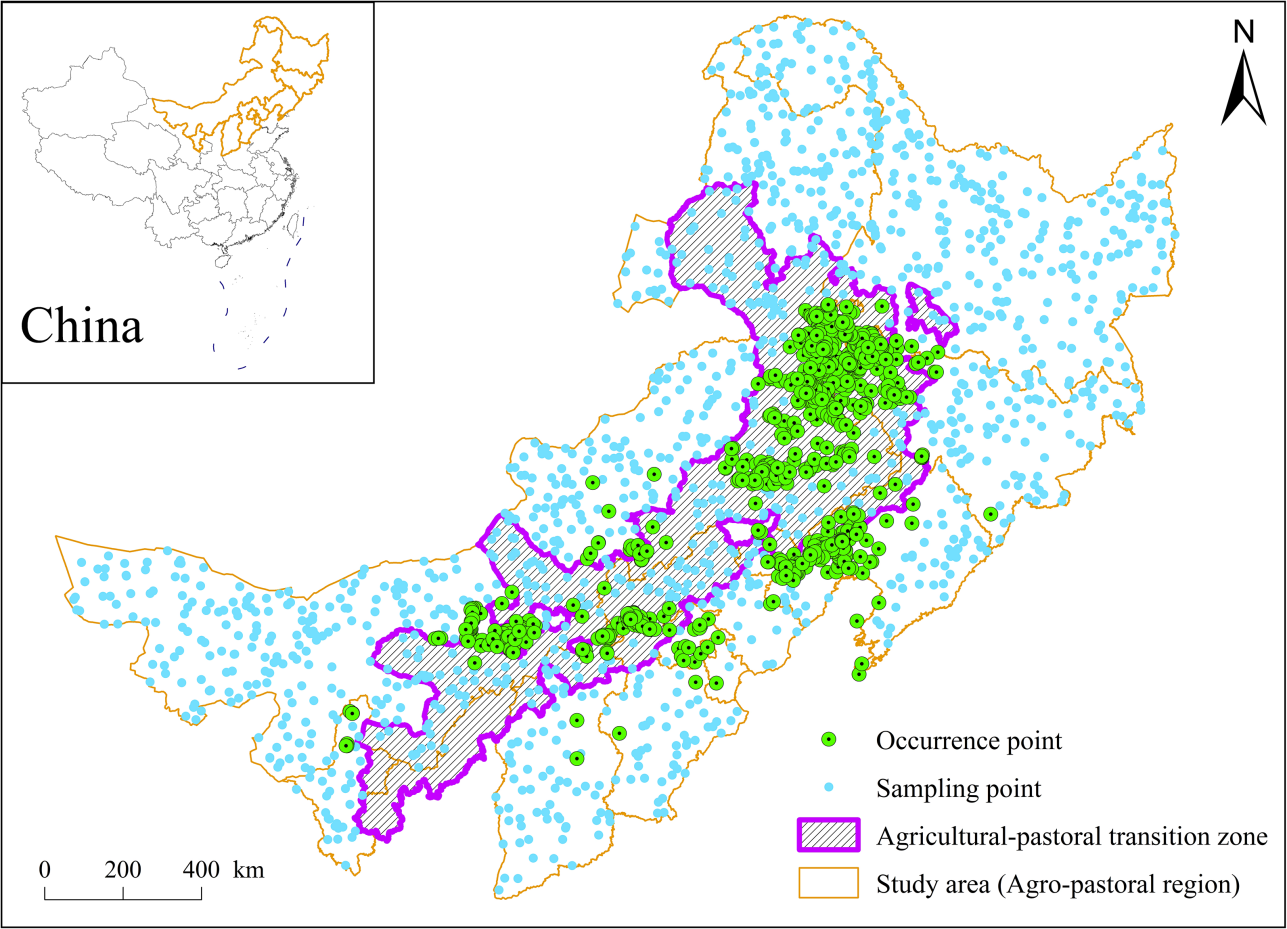
Spatial distribution of sampling and occurrence points of *Solanum rostratum.*.

The study was conducted in the Agro-Pastoral Region (APR) of northern China, a composite zone encompassing three distinct yet interconnected systems: agricultural regions (croplands), agricultural-pastoral transition zones (mixed farming-grazing areas), and pastoral regions (rangelands) (**Figure 1**). Spanning the Inner Mongolia Autonomous Region and parts of Liaoning, Jilin, Heilongjiang, Hebei, Shanxi, Shaanxi, Ningxia, and Gansu provinces, the APR represents a dynamic interface where intensive agriculture, transitional agro-pastoral practices, and nomadic pastoralism coexist. Climatically, the region transitions from a temperate monsoon climate in the eastern agricultural plains to a temperate continental arid/semi-arid climate in the western pastoral plateaus, with the transitional zone exhibiting intermediate conditions. Topographically, it includes plains, hills, mountains, and plateaus, further shaping heterogeneous land-use patterns.

The APR’s environmental vulnerability stems from its semi-arid to arid ecosystems, intensified by human activities such as cropland expansion, overgrazing, and infrastructure development. These pressures amplify risks of soil degradation, biodiversity loss, and invasive species establishment. Since its accidental introduction in 1981, *S. rostratum* has exploited the APR’s interconnected agricultural, transitional, and pastoral systems to spread rapidly, threatening both ecological stability and livelihoods. Therefore, this region, with its characteristic ecological transition, pronounced anthropogenic pressure, and the co-occurrence of all potential dispersal vectors, provides an ideal natural laboratory to test the hypothesis of multi-vector synergy driving patchy invasion patterns. By integrating these three subsystems, the APR serves as a critical model for studying multi-vector invasion mechanisms across ecological and anthropogenic gradients.

### 2.2 Mapping and characterizing spatiotemporal distribution patterns

#### 2.2.1 Phytogeographic data

To address the first objective of mapping spatiotemporal distribution of *S. rostratum*, we compiled occurrence records and location data from multiple sources, including government reports, herbarium archives, and peer-reviewed scientific literature (Wang et al., 2018). Field surveys conducted between 2021 and 2023 were used to validate and supplement these records. For locations lacking precise geographic coordinates, textual site descriptions were converted to decimal degree coordinates using a web-based geolocation tool, ensuring spatial consistency and accuracy. Only records with precise geographic coordinates or unambiguous location descriptions that could be reliably geocoded were retained. Coordinates for historical records with textual descriptions were obtained using the Google Geocoding API (Application Programming Interface). While spatial accuracy of early herbarium records varies, our validation and supplementation with recent field surveys (2021-2023) aimed to ensure a robust spatiotemporal dataset for analyzing distribution trends. Of the 1,520 sampling locations assessed, 1,234 were documented to the occurrence of *S. rostratum* populations (**Figure 1**).

#### 2.2.2 Spatial distribution and clustering dynamics analysis

We employed nearest neighbor analysis (NNA) to evaluate the spatial distribution patterns of *S. rostratum* occurrence and sampling points. This method calculates the average distance between each point and its nearest neighbor, comparing it to the expected distance under a random distribution model (Clark and Evans, 1954; Wang et al., 2006, 2019). The nearest neighbor index (NNI) quantified clustering tendencies, with an NNI value of 1 indicating a random distribution, where the observed distances between points matched the expected distances under a random spatial arrangement. Values below 1 (NNI< 1) signified a clustered distribution, reflecting greater point aggregation than would occur randomly, while values above 1 (NNI > 1) suggested a dispersed distribution, where points were more evenly spaced than predicted by chance.

To further assess spatial dynamics, we applied nearest neighbor hierarchical (NNH) clustering analysis, identifying hotspots—clustered patches where point densities significantly exceeded random expectations. Using ArcGIS 10.2, we delineated these patches and analyzed their temporal changes (number, size, and shape) at 5-year intervals since the species’ first record in China to 2023. This combined approach revealed shifts in invasion patterns, from initial clustering to potential dispersal or consolidation of hotspots over time.

#### 2.2.3 Spatial orientation and alignment of clustering patches relative to roads and rivers

The clustering patches derived from NNH analysis were approximated as ellipses. To assess the spatial association between patches and potential linear landscape features (roads and rivers), we first calculated the overlap rate for each feature type. A patch was considered to overlap with a road (or river) if its ellipse intersected with the nearest road (or river) segment. The overlap rate was defined as the percentage of patches showing such intersection.

The orientation of the major axis of each ellipse may indicate the primary direction of patch expansion. To identify potential landscape drivers of this expansion, we calculated the angle between the major axis of each patch and the nearest road or river segment. An angle of less than 45° was considered to indicate alignment, signifying that the patch’s extension direction was consistent with that of the road or river. Conversely, an angle of 45° or greater indicated no directional alignment. All angle calculations were performed in MATLAB R2020a (Mathworks Inc., Natick, MA, USA).

To assess whether the observed road-patch association could be an artifact of sampling bias (e.g., higher survey effort near roads), we performed two complementary analyses (detailed in **Supplementary Materials S1**, **S2**). First, we used inverse-probability weighting to recalculate overlap rates between patches and roads (or rivers), adjusting for variation in sampling intensity across accessibility classes. Second, we fitted a single-season occupancy model that explicitly separates detection probability (as a function of road accessibility) from true occupancy (as a function of distance to roads), thereby accounting for potential detection bias.

### 2.3 Simulating the wind-, vehicle- and animal- mediated dispersal

Consistent with the second research objective and the vector framework outlined in the Introduction, we aimed to quantify the most plausible dispersal vectors for *S. rostratum* in our study region. The dispersal syndrome of *S. rostratum* suggests that its seeds can be dispersed by water, wind, animals, and human activities (Cousens et al., 2008; Eminniyaz et al., 2013). Given the arid to semi-arid climate of the Agro-Pastoral Region where surface water flow is often limited and ephemeral, and based on preliminary field observations that did not suggest a dominant role of riparian corridors, we prioritized the quantification of wind-, vehicle-, and animal-mediated dispersal pathways. The potential role of hydrochory was evaluated a posteriori by analyzing the spatial relationship between identified patches and river networks (see Results 3.1).

#### 2.3.1 Wind dispersal modeling

Wind-mediated seed dispersal was estimated using a mechanistic simulation framework that integrates biological and wind parameters (Nathan et al., 2011). Biological parameters included seed sedimentation velocity (*v_t_*), average plant height (*h_t_*), fecundity (*β*), and seed release height probability (*p_r_*). Wind parameters comprised limiting wind speed (*U*), turbulence coefficient (*ĸ*), and vertical air velocity deviation (*σ_ω_*) (Caplat et al., 2012; Nathan et al., 2011). The parameters *σ_ω_* and seed release height probability *p_r_* were set according to the standard formulations in Nathan et al. (2011).

To obtain robust, region-wide estimates for the biological parameters, we employed a stratified random sampling strategy based on climate zones (Semi-arid Cold, Temperate Transitional, Semi-humid Warm) and land-use types (Cropland, Pastoral Rangeland, Agro-pastoral Ecotone) (Qin et al., 2005). From each of the nine resulting categories that contained *S. rostratum* patches, we randomly selected 3–5 representative patches (approximately 30% of patches per category) for field measurements. Within these patches, average plant height (*h_t_*) was measured for 50 randomly chosen individuals, and fecundity (*β*) was determined by destructively sampling 10 mature, fruit-bearing individuals per patch. Seed sedimentation velocity (*v_t_*) was determined experimentally by timing the fall of seeds released from heights of 0.5–2.0 m (100 replicates per height).

Wind data were obtained from the China Meteorological Data Service Center, including (1) China surface climate daily dataset (V3.0) (1951–2012) and (2) China surface climate standard value dataset (annual wind data) (1991–2020). For each occurrence location, mean wind speeds were calculated from the invasion year through 2020. To account for extreme conditions, we used empirical percentiles (95th, 99th, and 99.9th) of annual mean wind speeds derived from 71 meteorological stations in the study region (see **Supplementary Material S4** for details). Sensitivity of the model to the turbulence coefficient (*ĸ*) was also tested by varying *ĸ* from 0.3 to 0.5 (**Supplementary Material S4**).

#### 2.3.2 Modeling seed dispersal speed by vehicles

The alignment of *S. rostratum* clustering patches with roads suggests vehicle-mediated dispersal is a key mechanism. Vehicles facilitate long-distance seed transport via adhesion to tires or undercarriages, often creating linear dispersal patterns parallel to road networks (Christen and Matlack, 2009). To quantify this process, we integrated experimental simulations and mathematical modeling to assess seed adhesion dynamics across soil types and predict dispersal distances.

##### 2.3.2.1 Simulation experiment

We simulated seed adhesion dynamics across soil textures representative of the study region. Soil textures were systematically classified according to the USDA soil taxonomy system. The spatial distribution of these soil texture classes across *S. rostratum* habitats was georeferenced using data from the China Soil Database and the Second National Soil Survey, integrated and analyzed in R and GIS platforms. Each texture class was characterized by distinct particle-size fractions: sand (50-2000 µm), silt (2-50 µm), and clay (<2 µm). For each soil texture class, the adhesion experiment was replicated five times (n=5) to account for variability.

To replicate field conditions, soil slurries with particle-size distributions matching natural soils were prepared. For each trial, 100 inactivated seeds were uniformly scattered on the slurry surface. A standardized vehicle (model: medium-duty flatbed truck; speed: 70 km/h) was driven over the soil, after which residual seeds on the vehicle (wheels and mudguards) were collected. The adhesion rate was calculated as:


$$\text{Adhesion}\left(\%\right)=\frac{\text{Initial seeds}\left(100\right)-\text{Recovered seeds}}{\text{Initial seeds}\left(100\right)}\times 100$$

To assess distance-dependent seed retention, we tested seed loss at 13 distance intervals (0–4096 m). After each interval, dislodged seeds were counted, and the dispersal proportion was calculated as:


$$\text{Dispersal}\left(\%\right)=\frac{\text{Adhered seeds}-\text{Retained seeds}}{\text{Adhered seeds}}\times 100$$

##### 2.3.2.2 Modeling seed dispersal dynamics

Four exponential functions were tested to model seed drop-off rates (Bullock et al., 2011): (1) the simple exponential $f\left(d\right)=a^{{\text{e}}^{-bd}}$, assuming constant deposition; (2) the double exponential decreasing $f\left(d\right)=a{\text{e}}^{{\text{e}}^{-bd}}$; (3) the double exponential increasing $f\left(d\right)=a{\text{e}}^{-{\text{e}}^{bd}}$; and (4) the power exponential $f\left(d\right)=a{\text{e}}^{-d^{b}}$. Models were fitted to empirical data using Python, with selection based on maximizing *R*^2^ (goodness-of-fit) and minimizing *p*-values derived from 1,000 bootstrap iterations (Davison and Hinkley, 1997)). The optimal model was converted to a probability density function (PDF):


$$g\left(d\right)=N_{0}f\left(d\right)$$

where *N*_0_ represents the empirically derived initial seed count per patch. Dispersal distances were defined operationally as the 90th percentile distance (*d*_90_) under the PDF and the maximum distance (*d_max_*), calculated as the distance where cumulative *g*(*d*) ≥1, reflecting biological dispersal limits.

#### 2.3.3 Quantifying seed dispersal via animal-mediated epizoochory and endozoochory

Animals are critical vectors for invasive plant dispersal through epizoochory (external transport on fur) and endozoochory (internal transport via ingestion and defecation). For *S. rostratum*, prior work by Eminniyaz et al. (2013) demonstrated effective epizoochory via wool adhesion in sheep. Building on this foundation, our study quantifies both epizoochorous dispersal velocity and endozoochorous seed viability to holistically assess animal-mediated spread.

##### 2.3.3.1 Epizoochory: wool-adhesion dynamics and dispersal modeling

We extended Eminniyaz et al.’s framework by integrating two key parameters: (1) adhesion probability, defined as the proportion of fruits adhering to sheep wool, and (2) temporal retention, which quantifies the decline in adhered fruits over time. To model dispersal distances, we incorporated diurnal grazing patterns of domesticated sheep (*Ovis aries*), the dominant animal in the study region. Sheep movement data (average daily grazing distance and pattern) were obtained from published studies conducted in similar agro-pastoral systems of Inner Mongolia and from interviews with local herders (Hou et al., 2023; Zhang et al., 2025). Distance-dependent retention was modeled using four exponential decay functions (section 2.3.2), mirroring the vehicle-mediated dispersal framework. The adhesion and retention experiments were repeated three times (n=3) for each distance interval. The optimal function, selected by maximizing *R*^2^ (goodness-of-fit) and minimizing *p*-values, served as the dispersal kernel. From this, we derived two metrics: effective dispersal distance (*d*_90_), representing the 90th percentile of the probability density function, and maximum dispersal distance (*d_max_*), defined as the distance where cumulative seeds asymptotically approach or exceed 1.

##### 2.3.3.2 Endozoochory: seed viability post-ingestion

To assess endozoochory, we conducted feeding trials with six sheep (labeled A–F), each receiving 100 *S. rostratum* seeds mixed into forage. Feces were collected daily for seven days. For Sheep A, B and C, seeds were recovered via fecal washing to quantify survival rates. For Sheep D, E and F, feces were spread over nutrient-rich soil to test germination viability. One control group of 100 untreated seeds was planted in five pots under identical conditions. Germination rates for both experimental and control groups were recorded every five days to isolate the effects of ingestion on seed viability. A *post-hoc* power analysis was performed to assess the statistical power of this trial (**Supplementary Material S7**).

### 2.4 Identifying mechanistic drivers via speed comparisons

#### 2.4.1 Calculating the minimal arrival speed within and between the clustering patches

To investigate the dispersal mechanism of *S. rostratum* within patches, we conducted calculations to determine the minimal arrival speed based on empirical data. This approach estimates the minimum rate of spread required to explain the observed pattern of colonization, following the conceptual framework used to infer dispersal dynamics from occurrence records (Horvitz et al., 2017). We assumed that each occurrence point *k* has the potential to spread from other occurrence points *e* within the same patch that were identified prior to *k*. Within each patch *i*, our methodology involved the initial computation of the arrival speed between the occurrence point *k* and the other occurrence points *e* that occurred earlier than point *k*. Subsequently, we determined the minimum value among these calculated speeds as the minimum arrival speed of occurrence point *k* (Equation 1):

(1) $$v_{k}=\min((d_{e,k}/(t_{k}-t_{e}))|\forall e)$$

where *v_k_* is the minimum arrival speed of occurrence point *k*; *d*_(_*_e_*,*_k_*_)_ is the distance from the occurrence point *k* within patch *i* to other occurrence points *e* within the same patch; and *t_e_* and *t_k_* are the discovery times of occurrence point *e* and *k*, respectively, where *t_k_* > *t_e_*. ∀*e* indicates that all distribution points *e* discovered earlier than occurrence point *k* were considered in the calculation.

The minimum arrival speed within patch *i* was calculated as (Equation 2):

(2) $$v_{i}=\frac{1}{m}\sum_{k=1}^{m}v_{k},\;\left(k=1,2,\ldots ,m\right)$$

where *m* is the number of points *k* in patch *i*.

To further explore the minimum arrival speed between patches of *S. rostratum*, we calculated the minimum arrival speed from patch *j* to patch *i*, ensuring that the discovery time of earliest occurrence point within patch *j* preceded that of patch *i*. Subsequently, we selected the lowest value among these calculated speeds to determine the minimum arrival speed of *S. rostratum* (Equation 3):

(3) $$v_{i}=\min((d_{i,j}/(t_{j}-t_{i}))|\forall j)$$

where *v_i_* is the minimum arrival speed of patch *i* from patches *j*; *d_i_*,*_j_* is the distance between the earliest occurrence point of patch *i* and the occurrence point of patches *j*; *t_i_* is the discovery time of the earliest occurrence point of patch *i*; and *t_j_* is the discovery time of occurrence point of patches *j*. ∀*j* indicates that all patches *j* discovered earlier than patch *i* were considered in the calculation.

Both the minimal arrival speed within and between the clustering patches were calculated in MATLAB R2020a.

#### 2.4.2 Quantifying the contributions of wind, vehicle, and animal dispersal to the spread speed of *S. rostratum*

To quantify the effects of wind-, vehicle-, and animal-mediated dispersal on the actual minimum arrival speed (MAS) of *S. rostratum*, we first conducted exploratory data analysis (EDA). The MAS data (both intra- and inter-patch) exhibited a strongly right-skewed distribution (Shapiro-Wilk test, p< 2.2e-16) and did not meet the normality assumption of linear models, even after log-transformation (**Supplementary Material S8**, **Supplementary Figure 8**). Therefore, we selected a Gamma Generalized Linear Mixed Model (GLMM) with a log-link function, which is appropriate for positive, continuous, right-skewed data and obviates the need for transformation.

The response variable was the MAS for each occurrence point (for intra-patch analysis) or patch (for inter-patch analysis). Predictor variables were the estimated dispersal potential from each vector: wind potential (km/year, derived from the wind dispersal model in section 2.3.1), vehicle potential (km/year, derived from the adhesion and retention model in section 2.3.2), and animal (epizoochory) potential (km/year, derived from the wool-adhesion model in section 2.3.3.1). These continuous predictors were standardized (mean = 0, SD = 1) to improve model convergence and facilitate comparison of effect sizes. To account for the inherent spatial structure in the data—where points or patches closer in space may have more similar MAS values due to shared environmental conditions or dispersal history-we incorporated patch identity (patch_id) as a random intercept. This hierarchical structure explicitly models the non-independence of observations within the same spatial cluster (i.e., patch).

The full model was specified as: MAS ~ wind_potential_std + vehicle_potential_std + animal_potential_std + (1 | patch_id), family = Gamma (link = log).

Model fitting was performed using the glmer function in thelme4package in R. We also fitted a second model including all two-way and three-way interactions among the standardized vector potentials to test for synergistic effects.

Model performance was compared using Akaike’s Information Criterion (AIC) and R^2^ values (marginal R^2^ for fixed effects, conditional R^2^ for fixed plus random effects) calculated with the MuMIn package. The Gamma GLMM outperformed alternative models (Linear Mixed Model on raw or log-transformed data, Generalized Additive Mixed Model) based on AIC and R^2^ (see **Supplementary Material** for details). Variance Inflation Factors (VIFs) for all predictors were below 2, indicating no substantial multicollinearity. Model diagnostics (residual plots, DHARMa simulation-based tests) confirmed the adequacy of the Gamma distribution and log-link. Furthermore, to assess whether our model adequately accounted for spatial autocorrelation beyond the patch-level clustering, we examined empirical variograms of the Pearson residuals from the final model. No discernible spatial structure was detected in the residuals, supporting the assumption that the random effect of patch_id and the fixed effects of the dispersal vectors captured the major sources of spatial dependency in the response variable.

To quantify the relative importance of each dispersal vector, we performed variance decomposition using the LMG (Lindeman-Merenda-Gold) method (**Supplementary Material S9.1**). The LMG method decomposes the model R^2^ (here, the conditional R^2^ of the GLMM) into non-negative contributions from each predictor by averaging over all orderings of predictors, providing a robust and interpretable metric of predictor importance. The absolute contribution of each vector (and their interactions) was calculated as the proportion of the total variance explained (conditional R^2^) attributed to that term when added to a null model containing only the random effect. The relative contribution rate among the three main vectors was calculated from the LMG results.

To spatially illustrate the dominant dispersal mechanisms, we classified each patch into one of seven categories based on the predicted contributions of each vector. For each patch *i*, we calculated the relative contribution of each vector to the predicted MAS using the GLMM coefficients and the patch-specific average standardized potential for each vector. A vector was classified as dominant if its relative contribution exceeded 60%; as major if between 40-60%; as minor if between 20-40%; and as negligible if below 20%. Patches were then categorized as: (1) Animal-mediated (animal dominant, others negligible), (2) Vehicle-mediated (vehicle dominant, others negligible), (3) Wind-mediated (wind dominant, others negligible), (4) Wind × Animal (both wind and animal major, vehicle negligible), (5) Wind × Vehicle (both wind and vehicle major, animal negligible), (6) Vehicle × Animal (both vehicle and animal major, wind negligible), and (7) Wind × Vehicle × Animal (all three vectors major). This classification provides an intuitive spatial mapping of the primary dispersal drivers. To assess the robustness of this classification to the arbitrary choice of thresholds (dominant >60%, major 40-60%), we performed a sensitivity analysis by varying both the dominance (50-70%) and major (35-45%) thresholds (**Supplementary Material S9.2**).

## 3 Results

The application of the integrated methodology yielded three sets of key findings, corresponding directly to our research objectives. First, we characterize the spatiotemporal distribution patterns of *S. rostratum* invasion. Second, we present the quantified dispersal potential of wind, vehicle, and animal vectors. Finally, we synthesize these findings to identify the primary mechanisms driving both local and long-distance spread.

### 3.1 Spatiotemporal distribution and patchy dynamics

**Figure 2:**
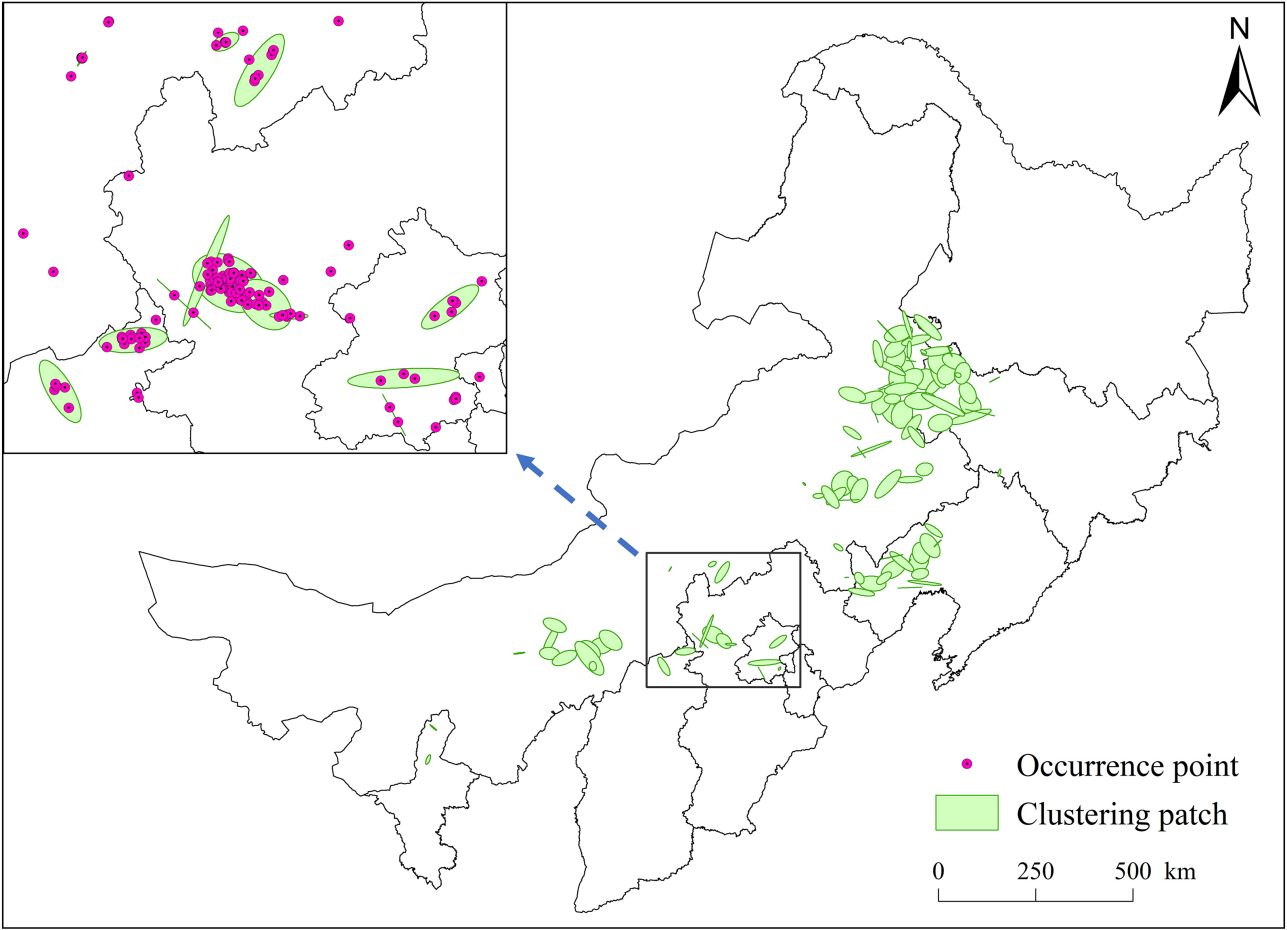
Spatial clustering patches of occurrence points of *Solanum rostratum*.

Nearest neighbor analysis (NNA) revealed contrasting spatial patterns between sampling and occurrence points of *S. rostratum*. Sampling points exhibited a random distribution (nearest neighbor index, NNI = 1.03; *P* < 0.01), with observed and expected nearest neighbor distances of 20.76 km and 20.19 km, respectively. In contrast, occurrence points showed significant clustering (NNI = 0.25; *P* < 0.01), with an average nearest neighbor distance of 5.50 km compared to the expected 22.24 km under randomness. Hierarchical clustering identified 103 distinct patches (**Figure 2**), encompassing 92% of all occurrence points (1,133 points), confirming a discontinuous, patchy invasion pattern.

**Figure 3:**
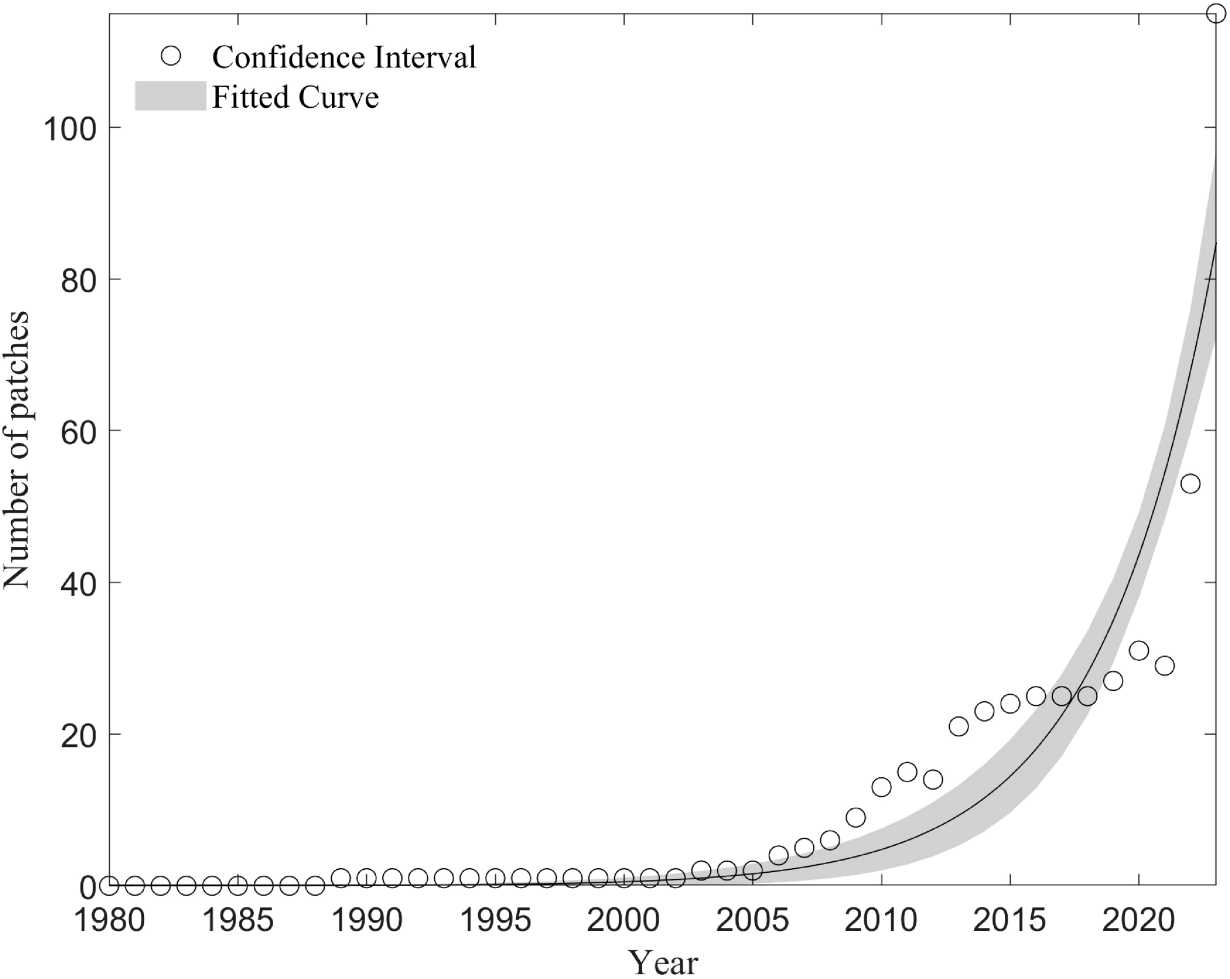
The number of clustering patches over time.

**Figure 4:**
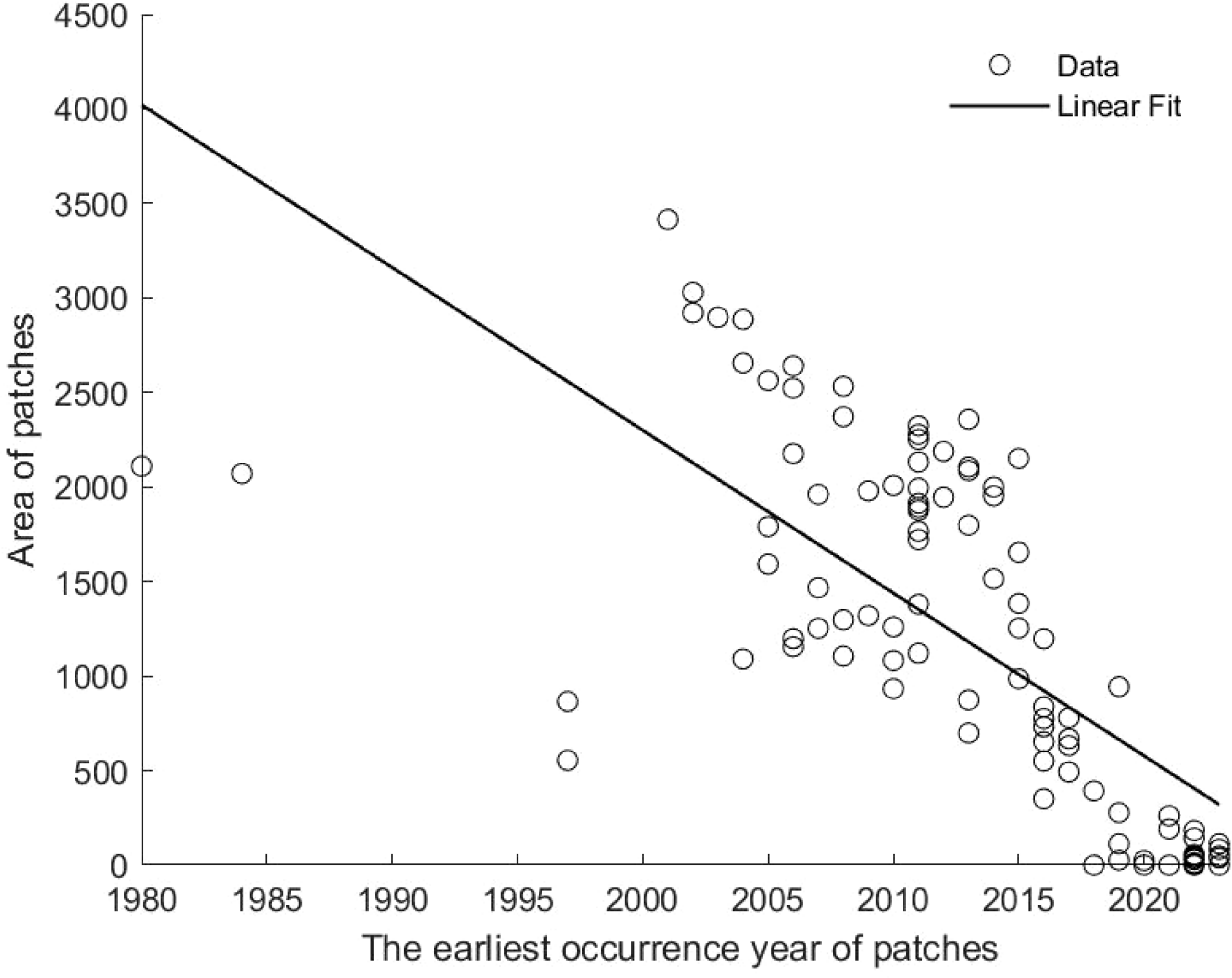
The correlation between the patches area and the discovery time of the earliest occurrence points within the patches.

The number of patches increased over time, delineating three invasion phases (**Figure 3**). From 1981-1988, only isolated occurrence points were recorded. Between 1989-2002, localized patch consolidation occurred, with one persistent cluster expanding in area. Post-2003, patch numbers surged, accompanied by both intra-patch growth and inter-patch colonization. Patch size (0.01-3,415.31 km^2^) correlated negatively with discovery year (P< 0.01), indicating earlier-established patches were larger (**Figure 4**).

Spatial orientation analysis revealed 94.64% of patches (97/103) overlapped with roads (**Supplementary Material S1**, **Supplementary Table 1C**), with their major axes aligned closely (mean angle ≈4.16°) to road networks (**Supplementary Material S3**, **Supplementary Figure 3A**). By contrast, only 13.39% of patches (14/103) overlapped rivers (**Supplementary Material S1**, **Supplementary Table 1C**), and their major axes showed no directional alignment (mean angle ≈54.28°; **Supplementary Material S3**, **Supplementary Figure 3B**), suggesting roads, not rivers, drive patch expansion.

To rule out the possibility that these associations were artifacts of sampling bias (e.g., higher survey effort near roads), we performed inverse-probability weighting and occupancy modeling (**Supplementary Materials S1**, **S2**). Sampling intensity was nearly constant across road accessibility classes (≤2.5% difference), and weighted overlap rates (94.68–94.72%) were virtually identical to the raw rate (94.64%) (**Supplementary Material S1**, **Supplementary Table 1C**). The occupancy model, which separates detection probability from true occupancy, revealed a strong negative effect of road distance on occupancy (coefficient = -0.5897, SE = 0.0655, P< 0.0001), while the detection covariate (accessibility) was only marginally significant (P = 0.066) (**Supplementary Material S2**, **Supplementary Table 2A**). These results robustly confirm that the road-patch association reflects actual ecological processes rather than sampling or detection artifacts.

### 3.2 Dispersal potential of individual vectors

**Figure 5:**
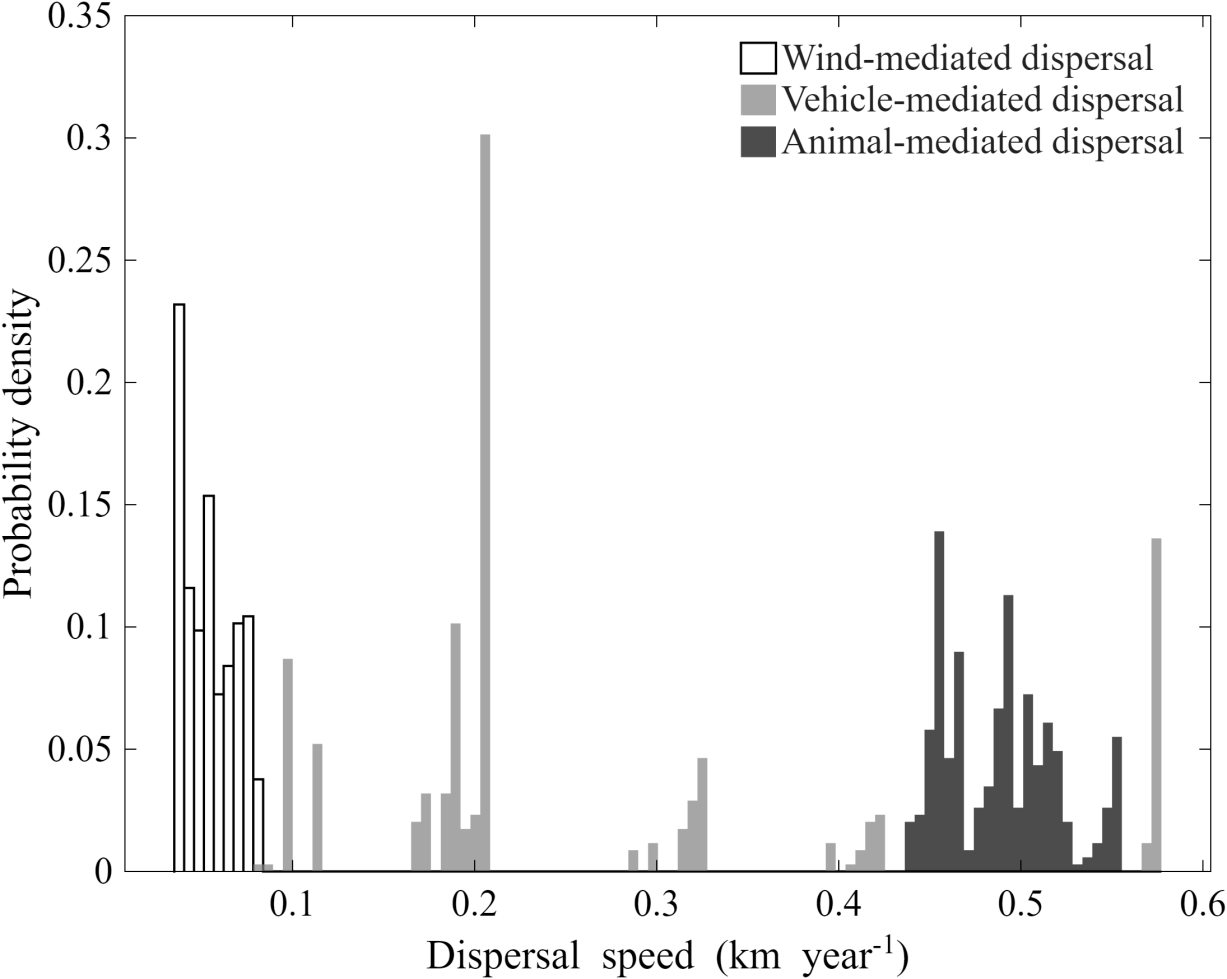
Modelled dispersal speeds of *Solanum rostratum* seeds via wind-, animal-, and vehicle-mediated vectors.

Wind simulations under site-specific mean wind speeds estimated dispersal rates of 0.04-0.08 km/year (**Figure 5**). Under the empirically derived 99.9th percentile wind speed (5.89 m/s), the modelled annual seed dispersal speed increased only marginally to a mean of 0.0976 km/year (range 0.0758-0.121 km/year; **Supplementary Material S4**, **Supplementary Table 4A**; **Supplementary Figure 4A**). Varying the turbulence coefficient (ĸ) from 0.3 to 0.5 changed the dispersal speed by less than 9% (**Supplementary Material S4**, **Supplementary Table 4B**). Even under these realistic extreme conditions, wind dispersal remained two orders of magnitude lower than vehicle- or animal-mediated dispersal.

Vehicle adhesion experiments demonstrated soil texture-dependent retention. Adhesion probabilities ranged from 0.07–0.30 across soil types, with clay-rich soils retaining seeds most effectively (**Supplementary Material S5**, **Supplementary Figure 5A**). The average retention ratios of seeds on vehicle surfaces across progressive transport distances are shown in **Supplementary Figure 5B** (**Supplementary Material S5**). All models (Eq. 4-7) showed significant explanatory power (P< 0.05; **Supplementary Figure 5C-S5F**), though fit quality varied. Based on optimal performance, Eq. 4 was selected to model seed attachment decay over distance. Maximum dispersal speeds for vehicle-adhered *S. rostratum* seeds varied regionally (0.08-0.58 km/year; **Figure 5**).

Epizoochory simulations indicated sheep could disperse seeds up to 4.80 km during 8-hour grazing, but retention decreased exponentially (92% at 0.60 km vs. 2% at 4.80 km; **Supplementary Material S6**, **Supplementary Figure 6A**). A double exponential model best fit retention dynamics (R^2^ = 0.955) (**Supplementary Material S6**, **Supplementary Figure 6B**). Using the best-fit decay function, we fitted the distance traveled by sheep after initially carrying fruits. Dispersal speed varied (0.44-0.56 km/year) with patch-specific *S. rostratum* fruit production (251–491 fruits/plant) through differential wool adhesion (**Figure 5**). Endozoochory trials detected no germination from seeds recovered from any of the six sheep (0% germination vs. 27.2% in controls). A *post-hoc* power analysis (**Supplementary Material S7**) indicated that the experiment can only rule out true survival rates >39.3%; lower levels of endozoochory cannot be statistically excluded. Therefore, while sheep gut passage severely reduces seed viability, a low probability of endozoochory remains possible.

### 3.3 Mechanistic drivers of observed spread speed

**Figure 6:**
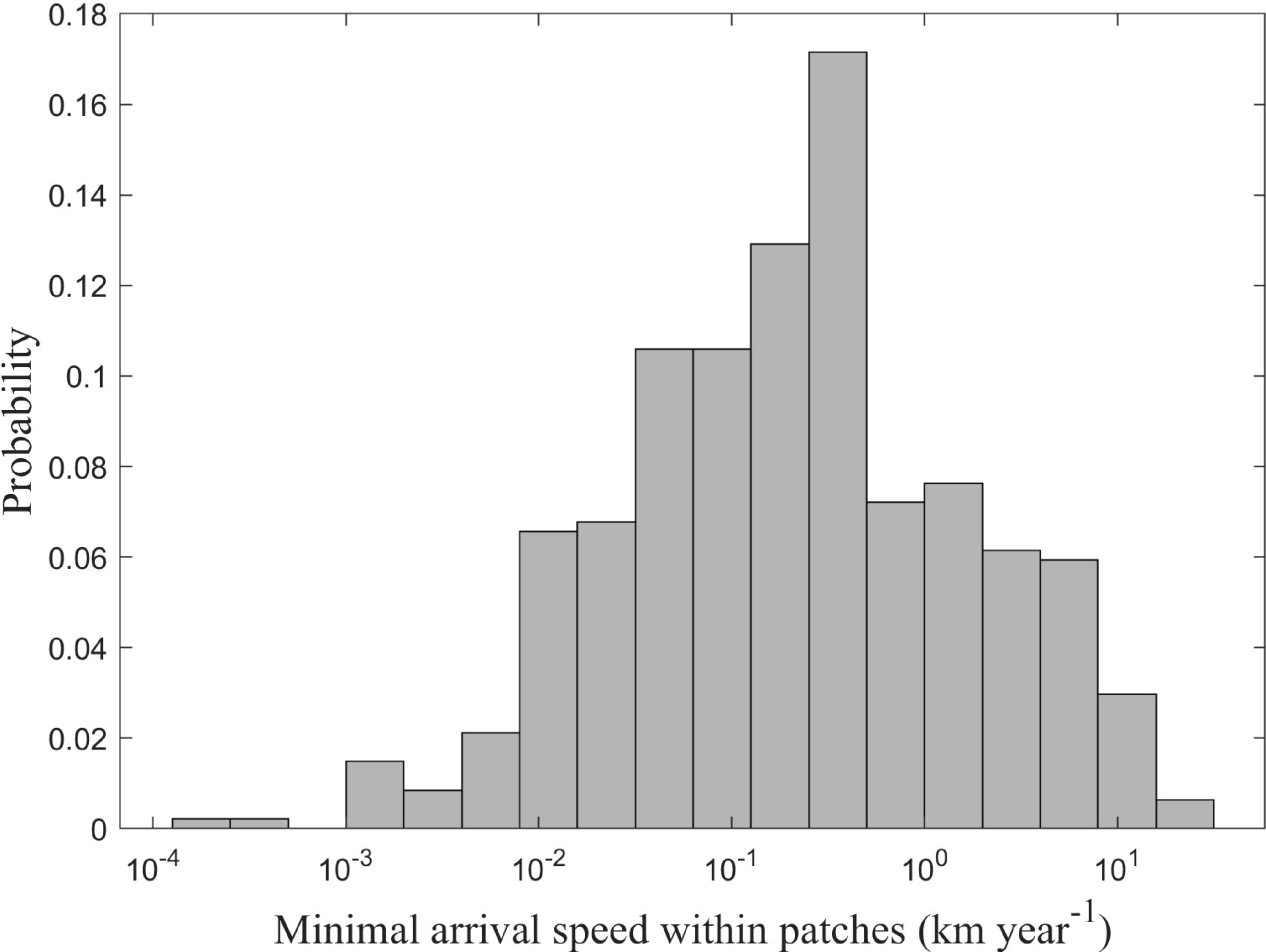
Frequency distribution of minimal arrival speed within clustering patches for *Solanum rostratum*.

**Figure 7:**
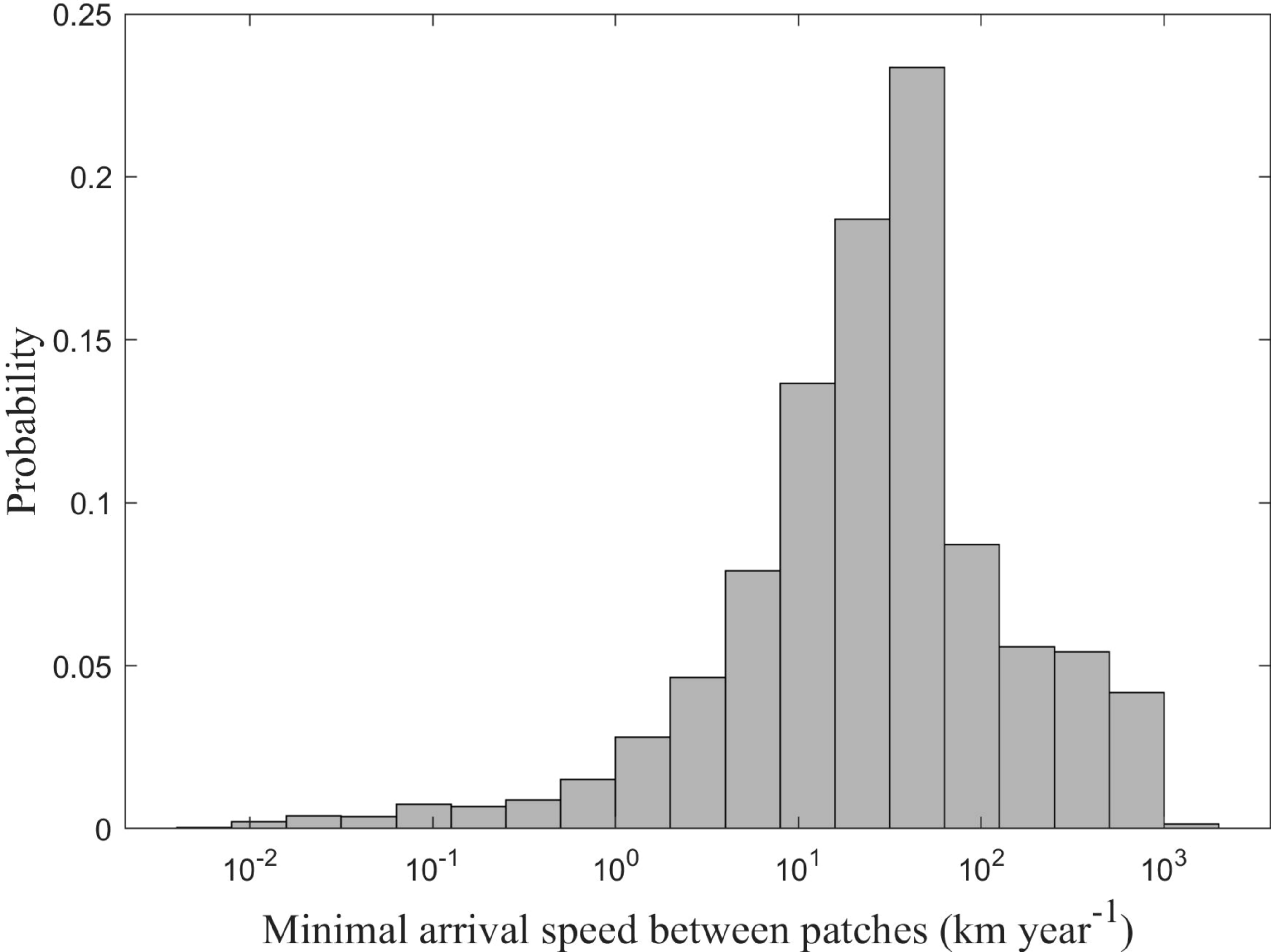
Frequency distribution of minimal arrival speed between clustering patches for *Solanum rostratum.*.

The calculated minimum arrival speed within patches ranged from 0.02 to 28.12 km/year, with a peak frequency at 0.81 km/year (**Figure 6**). In stark contrast, MAS between patches spanned 0.12 to 1,746.20 km/year, with a peak at 39.81 km/year (**Figure 7**). Inter-patch speeds were, on average, two orders of magnitude greater than intra-patch speeds.

To explain the variance in MAS, a Gamma GLMM was fitted. The full model (including vector interactions) explained 83.5% of the total variance (conditional R^2^). The fixed effects of the three vectors alone (without interactions) accounted for 59.3% of the variance (marginal R^2^), and including the random effect of patch identity increased the explained variance to 64.3% (conditional R^2^ for the main-effects model). Vehicle-mediated dispersal made the strongest single-vector contribution to explaining MAS (relative contribution: 55.3%), followed by animal-mediated epizoochory (43.3%) and wind (1.4%) (**Table 1**).

**Table 1:** Contribution rates and significance of dispersal vectors on the spread speed of *Solanum rostratum.*

Individual vectors	LMG contribution (R^2^)	Relative contribution rate (%)
Animal-mediated (epizoochory)	0.187	43.3
Vehicle-mediated dispersal	0.239	55.3
Wind-mediated dispersal	0.0062	1.43

Critically, synergistic interactions among vectors significantly amplified the explained variance. Two-way interactions were substantial (Wind × Vehicle: 33.48%; Wind × Animal: 32.35%; Vehicle × Animal: 29.05%), and the three-way interaction also contributed meaningfully (5.12%) (**Table 2**). This indicates that combined vector pathways are key to explaining spread rates.

**Table 2:** Contribution rates and significance of interaction effects on the spread speed of *Solanum rostratum.*

Interaction effects	Contribution rate (%)
Wind × Vehicle	33.48
Wind × Animal	32.35
Vehicle × Animal	29.05
Wind × Vehicle × Animal	5.12

Spatial classification of patches based on the dominant predicted vector(s) revealed distinct patterns (**Table 3**). Animal-mediated dispersal was the most frequent sole driver (40.0% of patches). Synergistic mechanisms were common, with Vehicle × Animal (22.2%) and the three-way interaction (17.8%) being prevalent. No patches were dominated by wind alone, indicating its role is primarily in combination with other vectors. A sensitivity analysis, varying the classification thresholds (dominant 50-70%, major 35-45%), confirmed that the assignment of patches to dispersal mechanism categories was robust, with an average concordance of 79.7% across all threshold combinations (**Supplementary Material S9.2**).

**Table 3:** Patch classification by dominant dispersal mechanism(s).

Dispersal mechanism	Number of patches	Percentage (%)	Mean MAS
Animal-mediated dispersal (epizoochory)	36	40.0	0.585
Vehicle × Animal	20	22.2	1.580
Wind × Vehicle × Animal	16	17.8	1.279
Vehicle-mediated dispersal	13	14.4	0.435
Wind × Vehicle	3	3.3	1.540
Wind × Animal	2	2.2	0.320
Wind-mediated dispersal	0	0.0	NA

## 4 Discussion

Our integrated analysis reveals that the patchy invasion of *Solanum rostratum* in northern China’s agro-pastoral region is driven by two distinct sets of mechanisms operating at different spatial scales. At the local scale, within-patch expansion is primarily governed by the synergistic interaction of multiple natural and anthropogenic vectors-wind, animal epizoochory, and vehicular transport. At the regional scale, the long-distance establishment of new patches exceeds the capacity of these combined local vectors, implicating human-mediated jump dispersal as the critical driver of range expansion. The following sections interpret these key findings in the context of invasion ecology and integrated pest management.

### 4.1 Spatio-temporal distributional patterns of Solanum rostratum

Understanding the spatiotemporal patterns of non-native species is of both theoretical and practical importance (Christoph, 2017; Petrovskaya et al., 2017). For species with a continuous distribution, eradication efforts can begin at their edges and proceed counter to their direction of expansion. In contrast, species with a patchy distribution require targeted eradication focused on identifying discrete, dense populations. Unlike continuous-front distributions, patchy patterns are spatially heterogeneous and thus demand more strategic resource allocation (Petrovskii et al., 2005; Petrovskaya and Zhang, 2020). Our findings revealed that the occurrence points of *S. rostratum* exhibited a clustered (non-random) distribution pattern, contrasting with the random distribution observed at sampling points. Among 1133 occurrence points, 103 clustered patches were identified, accounting for 92% of all occurrences. This confirms a patchy distribution pattern. Additionally, we determined the emergence time of each patch and documented temporal variations in patch size.

Although *S. rostratum* was first recorded in China in 1981, the earliest clustered patch was not observed until 1988, with multiple patches emerging only after 2003. Analysis of patch areas revealed a significant negative correlation between patch emergence time and size, indicating that earlier patches tended to be larger. This highlights two dispersal modes for *S. rostratum* in China: short-distance continuous spread and long-distance cross-regional expansion. Prior to 2003, spread was largely confined within existing patches. Post-2003, expansion occurred both within and between patches, reflecting a complex pattern of intra- and inter-regional spread. This shift may be linked to increased human trade activities in the 21st century, which likely facilitated rapid invasion (Reino et al., 2017; Sardain et al., 2019; Takahashi and Park, 2020).

Spatial analysis of clustered patches showed that 97 patches (94.64%) overlapped with roads, with their long axes aligned parallel to road segments. This strong spatial association suggests that roads likely serve as primary corridors facilitating patch expansion direction, although we cannot fully rule out potential sampling bias due to higher accessibility and detection probability along roads (Auffret et al., 2014). In contrast, 14 patches (13.39%) overlapped with rivers, but the angles between their long axes and river courses indicated no directional alignment. These results strongly suggest that human activities, particularly road networks, are a primary driver of dispersal direction at the landscape scale. In contrast, the lack of directional alignment with rivers, despite some spatial overlap (13.39% of patches), indicates that fluvial corridors are not a major vector for directed spread in this system. The observed overlap may instead reflect shared habitat preferences of *S. rostratum* for riparian zones or simply coincidental distribution, rather than active hydrochory (Banks et al., 2015; Wichmann et al., 2009). This strong spatial association between patches and road networks is consistent with, and provides strong circumstantial support for, our *a priori* hypothesis that vehicular transport serves as a primary dispersal vector at the landscape scale (see Introduction). Critically, this correlative pattern was subsequently validated and mechanistically explained through direct evidence. Our vehicle adhesion experiments quantified the seed transport potential under realistic soil conditions (Section 3.2), demonstrating a viable physical pathway. Furthermore, the Gamma Generalized Linear Mixed Model (GLMM) statistically attributed the largest single-vector contribution (55.3%) to the variance in minimum arrival speed to vehicle-mediated dispersal (Section 3.3; **Table 1**). Thus, the convergence of evidence—from spatial pattern analysis, controlled experimental simulation, and variance-partitioning statistical modeling—robustly confirms the causal role of vehicular transport in driving patch orientation and expansion. This multi-method approach moves the inference beyond initial spatial correlation to established mechanistic causation.

### 4.2 Localized spread of Solanum rostratum within patches: a synergy of wind, animal, and vehicular dispersal

The patchy distribution pattern of *S. rostratum* arises from a complex interplay of dispersal mechanisms. Our findings reveal that this invasive species exhibits considerable variation in minimum arrival speeds, ranging from 0.02–28.12 km/year within patches and 0.12–1,746.20 km/year between patches, with peak probabilities at 0.81 km/year and 39.81 km/year, respectively. This broad range reflects the species’ capacity for both localized expansion through short-distance dispersal and regional spread via long-distance vectors (Hengeveld, 1989), consistent with patterns observed in other invasive plant species (Nathan, 2006; Wang et al., 2011). While long-distance dispersal drives regional colonization, localized spread within patches is mediated by three key vectors: wind, animals, and human transportation networks.

Wind-mediated dispersal contributes to short-distance seed movement but shows limited spatial scalability. Eminniyaz et al. (2013) conducted a field experiment to investigate wind dispersal of *S. rostratum*, marking plant branches with red string to track dispersal distances. Their results showed that under typical wind conditions in Changji City, Xinjiang Uygur Autonomous Region, branches dispersed 3.4 ± 0.8 m (range: 0.1–14.9 m). However, these measurements reflect branch dispersal and may not represent seed dispersal dynamics. We found that seed-bearing burs exhibit distinct behavior: when seeds mature, burs open, releasing most seeds. Using site-specific mean wind speeds, we estimated wind-mediated seed dispersal rates of 0.04–0.08 km/year across patches. We acknowledge that seed terminal velocity was measured under laboratory conditions. However, such still-air measurement is a standard, validated approach in mechanistic wind dispersal research (Nathan et al., 2011; Kuparinen, 2006). Our model explicitly incorporates turbulence parameters (*κ* and σ*_ω_*) that bridge laboratory-field gaps, and sensitivity analysis shows minimal response to their variation (**Supplementary Material S4**). Regional wind speed heterogeneity likely drives variation in dispersal distances, yet wind alone cannot explain the species’ rapid expansion to Liaoning, Inner Mongolia, Hebei, and 10 other provinces. This discrepancy points to the critical role of anthropogenic vectors in bridging localized and regional dispersal.

Road networks emerged as the dominant landscape feature influencing *S. rostratum* distribution, with 92% of clustering patches overlapping roadways and expansion axes strongly aligned with road orientation. This contrasts sharply with river systems, where only 13.39% of patches showed spatial overlap and minimal directional correspondence (10% within 45° of river flow). Our vehicle adhesion experiments demonstrated seed transport distances of 80–577 m, with natural adhesion rates ≤30%—substantially lower than the >256 km retention distances reported in simulated adhesion experiments (Taylor et al., 2012). This highlights a key limitation of laboratory studies: realistic adhesion dynamics reduce vehicular dispersal efficiency but remain sufficient to facilitate patch-to-patch connectivity.

Animal vectors complement these mechanisms through intermediate-range dispersal. Sheep-mediated transport via wool adhesion showed velocities of 0.44–0.56 km/year, correlated with patch fruit production. However, ingestion experiments revealed a critical demographic constraint: while control seeds maintained 27.2% germination viability, none of the seeds recovered from sheep feces remained viable, consistent with global patterns of digestive-mediated seed viability loss (Eminniyaz et al, 2013). Nevertheless, our sample size (n = 6) is modest; a *post-hoc* power analysis (**Supplementary Material S7**) indicates that while the experiment can rule out high survival rates (>39.3%), it cannot statistically exclude a low probability of endozoochory.

The synergy of these vectors exceeds their individual contributions. Our quantitative analysis using a Gamma GLMM revealed that vehicle-mediated dispersal was the single most important vector in explaining variation in MAS (relative contribution: 55.3%), followed by animal-mediated epizoochory (43.3%) and wind (1.4%). Critically, the model incorporating interactions explained a greater proportion of variance, with two-way interactions among all vector pairs being particularly strong (each accounting for 29-33% of the explained variance attributable to interactions). The three-way interaction (5.12%) further demonstrates emergent dispersal patterns-e.g., wind depositing seeds near roads for vehicular transport, or animals moving seeds between habitat patches. The classification of patches into seven dispersal mechanism categories (**Table 3**) revealed that animal-mediated dispersal was the most common sole driver (40.0% of classifiable patches), while no patches were dominated exclusively by wind. Synergistic effects were prevalent, with Vehicle × Animal (22.2%) and Wind × Vehicle × Animal (17.8%) being the most common interaction types. The absence of wind-dominated patches suggests that wind acts primarily in combination with other vectors rather than as a sole dispersal agent. These findings support Auffret et al(2014) framework of synergistic vector acceleration in invasive spread while providing mechanistic insights into *S. rostratum* invasion dynamics. The differential contributions and interactions of these pathways not only explain patch-scale expansion rate variability but also mandate integrated management strategies targeting multiple vectors simultaneously.

### 4.3 Beyond wind, vehicles, and animals: human-mediated jump dispersal via contaminated straw drives Solanum rostratum’s patch-to-patch invasion

Our findings reveal that the minimum arrival speed (MAS) between patches spans from 0.12 km to 1,746.20 km per year, with a frequency distribution peak around 39.81 km per year. The MAS of *S. rostratum* between patches is significantly higher than minimum arrival speeds of alien species in China reported in some previous studies (e.g., Horvitz et al., 2014). This disparity can be attributed to differing computational frameworks. Traditional frameworks often rely on administrative units as the smallest unit of analysis to calculate dispersal speed across administrative boundaries. In contrast, our research adopted a more nuanced approach based on the actual spatial distribution patterns formed by occurrence points, computing dispersal speed between clustering patches. This methodology better captures rapid dispersal dynamics and suggests that cross-regional dispersal speed of alien plant species can exceed tens of kilometers.

Comparing the MAS of *S. rostratum* within and between patches reveals that the dispersal speed between patches is approximately 100 times faster than within patches. This notable difference suggests that dispersal between patches is influenced by mechanisms operating over greater distances. Even under the most extreme empirically derived wind speed (99.9th percentile, 5.89 m/s), the simulated seed dispersal speeds remained at least one order of magnitude lower than the observed dispersal speed between patches. This indicates that wind-mediated mechanisms cannot account for inter-patch spread. Similarly, the maximum dispersal distances via vehicle-mediated and animal-mediated pathways were substantially shorter than the higher range of inter-patch dispersal rates observed for *S. rostratum*. This quantitative disparity strongly implicates alternative long-distance dispersal vectors, particularly anthropogenic transport of contaminated materials.

Our field investigations revealed that occurrence points of *S. rostratum* in expansion frontier areas were predominantly found in backyard stockpiles of straw maintained by herdsmen. Furthermore, we observed that straw purchased by herdsmen from local grass markets is contaminated with *S. rostratum* seeds and branches. Thus, we suggest that the inter-patch dispersal of *S. rostratum* may occur frequently through the transportation of contaminated straw, leading to the colonization of new patches. While this observational evidence strongly supports the hypothesis, future studies incorporating traceability of agricultural material movement and quantitative seed load assessments are needed to conclusively establish this causal pathway and quantify its contribution relative to other anthropogenic vectors.

## 5 Conclusion

**Figure 8:**
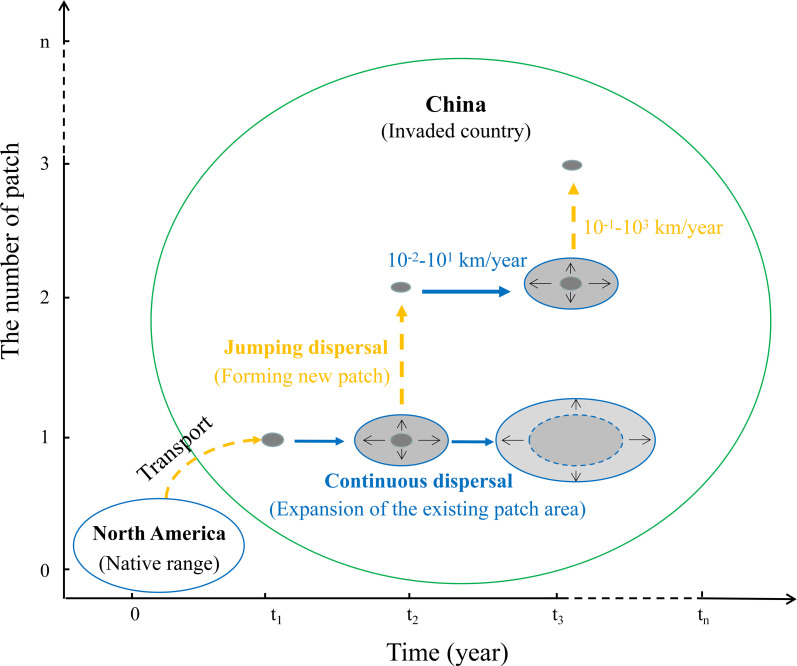
Conceptual representation of the patchy distribution and dual dispersal modes of *Solanum rostratum* in the Agro-Pastoral region of Northern China. The horizontal axis represents the timeline of the species’ invasion, while the vertical axis quantifies the number of patches over time. The native geographical range of *S. rostratum* is denoted by a blue-bordered, unfilled ellipse, whereas its invasive spread within China is demarcated by a green-bordered, unfilled ellipse. Clustering patches of *S. rostratum* (gray-filled ellipses with blue borders) indicate areas of concentrated growth. Within these clusters, local continuous dispersal (blue solid arrows) illustrates gradual expansion from existing patches without forming new ones, with a minimum arrival speed of 10^-2–^10^1^ km/year. In contrast, jumping dispersal (yellow dashed arrows) represents long-distance colonization between clusters, establishing new patches at a higher minimum arrival speed (10^-1–^10^3^ km/year), reflecting the species’ capacity for rapid, large-scale spread.

This study elucidates the dual mechanisms underpinning the patchy invasion of *S. rostratum* in northern China (**Figure 8**). At the local scale, spread within patches is driven by a synergy of wind, animal epizoochory, and vehicular dispersal, with the latter two being most influential. This multi-vector interaction explains the heterogeneous, yet relatively slower, expansion of existing infestations. In contrast, the rapid establishment of new, distant patches is driven by a separate, long-distance jump dispersal mechanism, most likely human-mediated transport of contaminated materials. These findings demonstrate that complex invasion patterns can arise from the interplay of different dispersal processes operating at distinct spatial scales. Effective management must therefore adopt a dual strategy: implementing local controls targeting synergistic vectors (e.g., vehicle cleaning, targeted grazing management) while simultaneously developing regional policies to intercept human-mediated jump dispersal pathways (e.g., inspection and cleaning of agricultural produce). This integrated, scale-aware approach is essential for mitigating the impact of this and similar invasive species in agro-pastoral ecosystems.

## Data Availability

The original contributions presented in the study are included in the article/**Supplementary Material**. Further inquiries can be directed to the corresponding authors.
