# Supplementary material for: Mechanisms underlying patchy distribution pattern and spread of the invasive species Solanum rostratum (Solanaceae) in the Agro-Pastoral region of Northern China
Source: Front Plant Sci. 2026 May 13;17:1809773. doi: 10.3389/fpls.2026.1809773 (PMC13212342; doi:10.3389/fpls.2026.1809773)
Supplement: Supplementary file 1 [file DataSheet1.docx]

**Supplementary Material**

S1 – Quantification of sampling bias and weighted overlap analysis

S2 – Occupancy model accounting for detection bias

S3 – Orientation of clustering patches relative to roads and rivers

S4 – Revised wind dispersal model

S5 – Vehicle‑mediated seed adhesion and retention dynamics

S6 – Animal‑mediated epizoochory: fruit retention on sheep wool

S7 – Power analysis for endozoochory (frequentist)

S8 – Distribution of minimum arrival speed (MAS) within and between patches

S9 – LMG variance decomposition and threshold sensitivity analysis

**S1 – Quantification of sampling bias and weighted overlap analysis**

To assess whether survey effort was biased towards roads or rivers, we quantified sampling intensity across accessibility classes for both landscape features and applied inverse-probability weighting to recalculate overlap rates. This analysis directly tests the robustness of the observed road-patch and river-patch associations to sampling bias.

**Methods**

A 10 km × 10 km grid was overlaid on the study area. For each grid cell, sampling intensity was calculated as the number of survey points per 100 km^2^. Cells were classified into three road‑accessibility classes based on distance to the nearest road (threshold schemes A, B, C; Table S1a shows scheme B as an example, with results for schemes A and C being very similar). The mean sampling intensity of each class was used to compute inverse-probability weights (w = 1 / mean intensity). Weighted overlap rates (proportion of patch-covered grid cells within 500 m of a road or river) were then recalculated and compared with raw (unweighted) rates. In addition, a weighted logistic regression was performed with patch presence as the binary response and distance to road (or river) as the predictor, using the same weights, to further assess the sensitivity of the road-patch association.

**Results**

Under scheme B, mean sampling intensity varied minimally across road accessibility classes: 0.02545 points/100 km² for high-accessibility cells (<300 m), 0.02606 for medium-accessibility cells (300-1500 m), and 0.02610 for low-accessibility cells (>1500 m) (Table S1a). Similarly, for rivers, intensities ranged from 0.02500 to 0.02611 across classes (Table S1b). Because sampling intensity was nearly constant, inverse-probability weights were close to 1. Consequently, weighted overlap rates for roads (94.68-94.72%) were virtually identical to the raw rate (94.64%), and for rivers (13.38-13.95%) closely matched the raw rate (13.39%) (Table S1c). Thus, sampling intensity was almost constant across road accessibility classes (≤2.5% difference), and weighted overlap rates were nearly identical to raw rates, indicating that the strong road–patch association is not an artefact of sampling bias.

The weighted logistic regression sensitivity analysis (Figure S1a) further confirmed these findings. For roads, the estimated coefficients for distance were consistently negative and statistically significant under all three weighting schemes (A, B, C), indicating that closer proximity to roads is associated with a higher probability of patch presence-a positive effect of road proximity. For rivers, the coefficients were small and not significant, showing no consistent effect. Taken together, the strong road-patch association is robust to sampling bias, whereas rivers showed no consistent spatial association with patches.

**Table S1a** Sampling intensity by road accessibility class (scheme B)

| Accessibility class | Distance to road (m) | Number of grid cells | Mean sampling intensity (points/100 km²) |
| --- | --- | --- | --- |
| High | <300 | 66 | 0.02545 |
| Medium | 300–1500 | 193 | 0.02606 |
| Low | >1500 | 1191 | 0.02610 |

*Note: Results for schemes A and C were very similar and are not shown for brevity.*

**Table S1b** Sampling intensity by river accessibility class (scheme B)

| Accessibility class | Distance to river (m) | Number of grid cells | Mean sampling intensity (points/100 km²) |
| --- | --- | --- | --- |
| High | <300 | 8 | 0.02500 |
| Medium | 300–1500 | 18 | 0.02611 |
| Low | >1500 | 1424 | 0.02607 |

**Table S1c** Raw and weighted overlap rates for roads and rivers

| Scheme | Road raw (%) | Road weighted (%) | River raw (%) | River weighted (%) |
| --- | --- | --- | --- | --- |
| A | 94.64 | 94.68 | 13.39 | 13.95 |
| B | 94.64 | 94.72 | 13.39 | 13.38 |
| C | 94.64 | 94.72 | 13.39 | 13.70 |


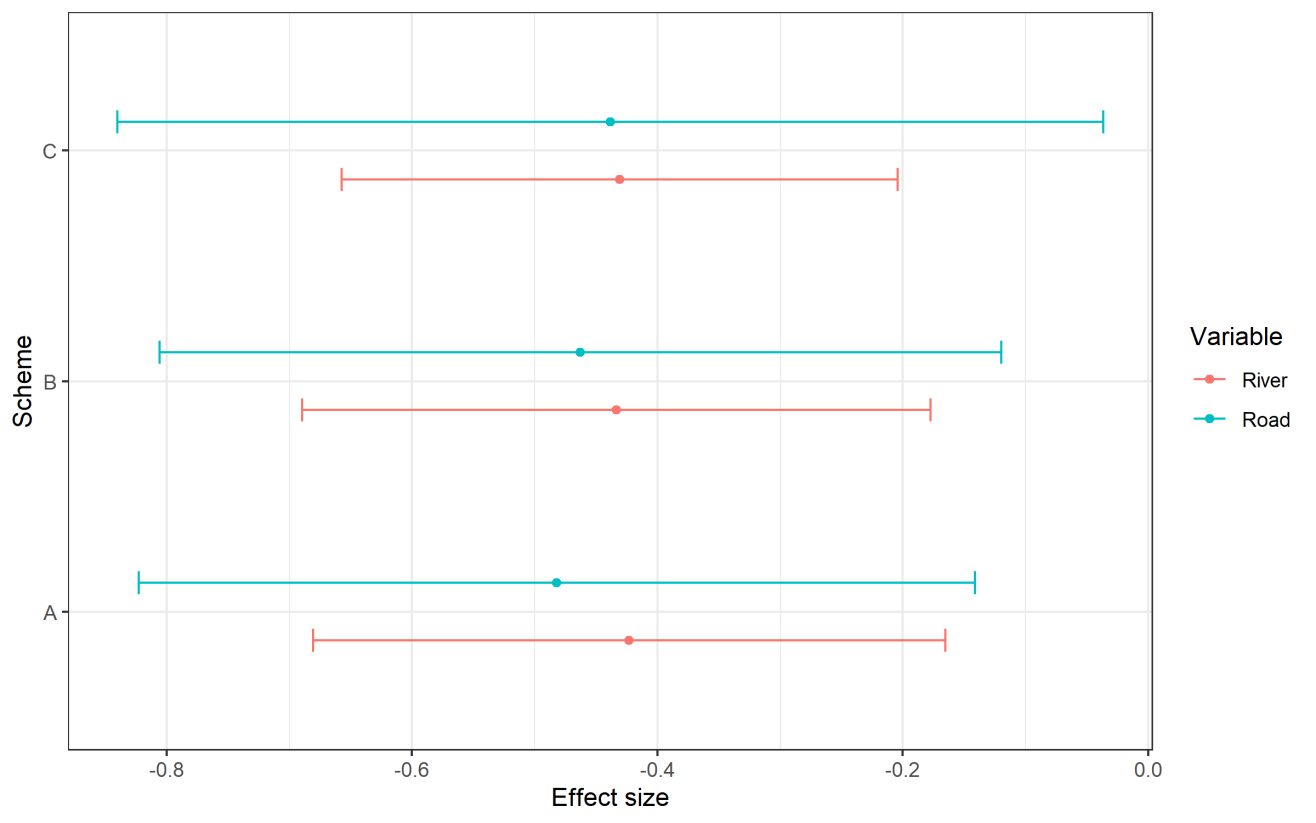


**Figure S1a** Forest plot of road and river effects from weighted logistic regression (sensitivity analysis)

**S2 – Occupancy model accounting for detection bias**

Even when sampling intensity is balanced (as shown in S1), detection probability per survey occasion may still differ between road‑proximal and road‑distant sites because plants near roads are more visible to observers. Such detection bias could confound the estimated effect of road distance on occupancy. To separate detection from occupancy, we fitted a single‑season occupancy model with road accessibility as a covariate in the detection submodel (MacKenzie et al., 2002). This analysis tests whether the negative association between road distance and patch occurrence persists after correcting for detection bias.

**Methods**

A single‑season occupancy model was fitted using the unmarked R package. Detection histories with at least three survey years were constructed for a 10 km grid. The detection process was modelled as logit(p) = α₀ + α₁·accessibility, where accessibility = log(dist_road + 10) (standardized). The occupancy process was modelled as logit(*ψ*) = β₀ + β₁·dist_road (standardized). The model converged after 2000 iterations. Residual spatial autocorrelation was assessed using Moran’s I on the residuals.

**Results**

The occupancy model revealed a strong negative effect of road distance on occupancy probability after accounting for detection bias. The coefficient for standardized distance to road in the occupancy submodel was -0.5897 (SE = 0.0655, P < 0.0001), indicating that occupancy probability declines sharply as distance from roads increases (Table S2a, Figure S2a). The detection covariate (accessibility) showed a marginally non-significant positive effect (estimate = 0.0912, SE = 0.0496, P = 0.066), suggesting a weak tendency for higher detection probability near roads, but this effect did not reach conventional significance. Critically, the strong and highly significant effect of road distance on occupancy remained after including this detection covariate. Residual spatial autocorrelation was significant (Moran’s I, P = 1.44 × 10^-11^), indicating some remaining fine-scale spatial structure not captured by the model, likely due to unmeasured environmental variables. Nevertheless, the effect of road distance is large, highly significant, and robust. These results confirm that after correcting for detection bias, roads remain a genuine driver of patch distribution.

**Table S2a** Coefficient estimates of the occupancy model

| Parameter | Estimate | SE | P‑value |
| --- | --- | --- | --- |
| Detection: accessibility | 0.0912 | 0.0496 | 0.066 |
| Occupancy: distance to road (standardized) | -0.5897 | 0.0655 | <0.0001 |


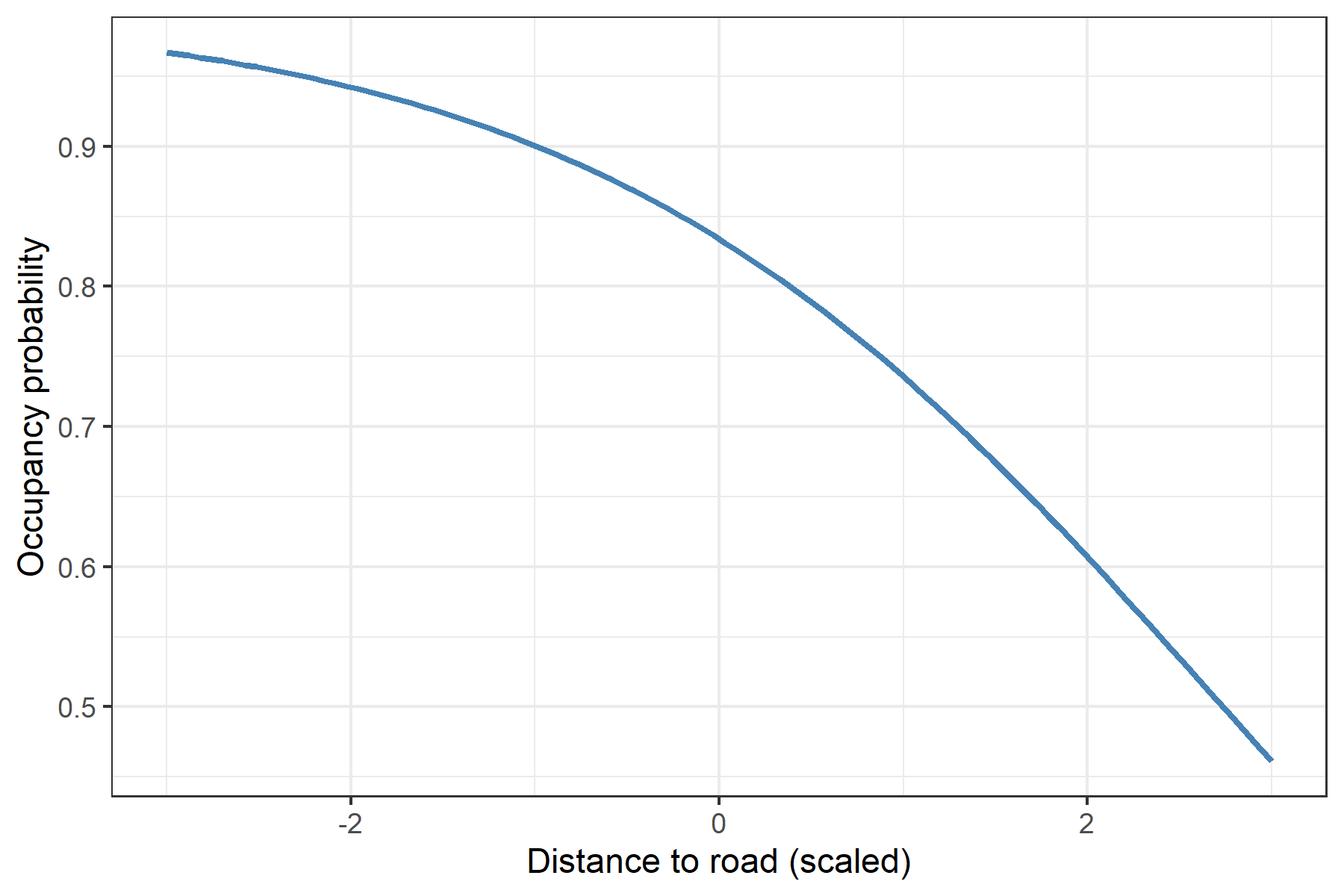


**Figure S2a** Predicted occupancy probability as a function of standardized distance to road

**S3 – Orientation of clustering patches relative to roads and rivers**

To provide a detailed visualisation of orientation of clustering patches relative to roads and rivers, we present frequency histograms of the angles between the major axis of each patch and the nearest road or river segment.

**Methods**

The angle between the major axis of the ellipse and the nearest road segment (or river segment) was calculated in MATLAB R2020a. Angles were binned into 10° intervals from 0° to 90°. An angle <45° was considered alignment, while ≥45° indicated no directional alignment. The frequency distributions were plotted as histograms.

**Results**

The angle between patch major axes and roads showed a strong peak near 0° (mean ≈4.16°; Figure S3a), confirming that patch expansion direction is closely aligned with road orientation. In contrast, the angles between patch axes and rivers were broadly distributed, with a mean of ≈54.28° (Figure S3b). These results support the conclusion that roads, not rivers, are the primary landscape corridors directing patch expansion.


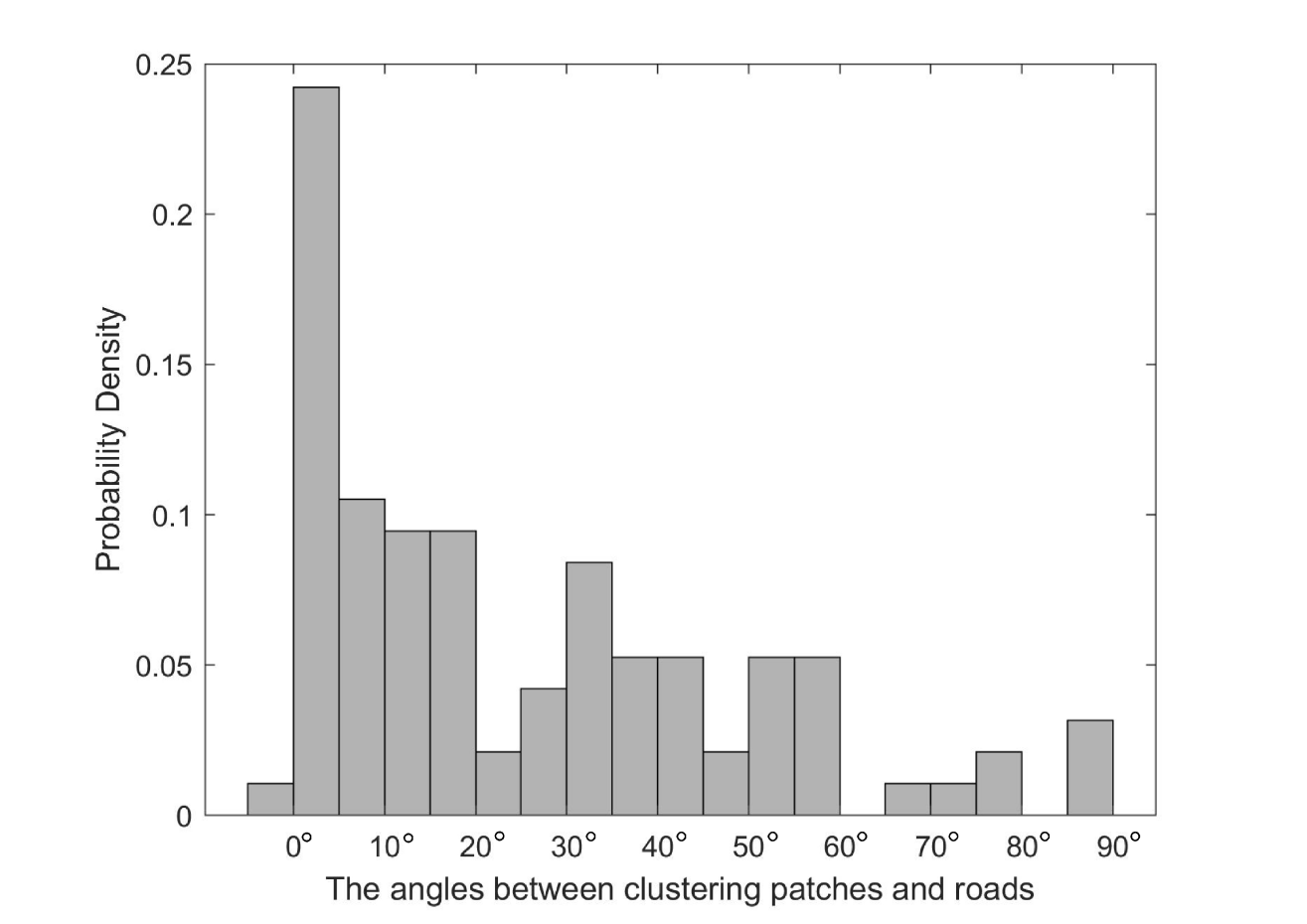


**Figure S3a** Frequency distribution of the angles between the long axis of clustering patches and roads


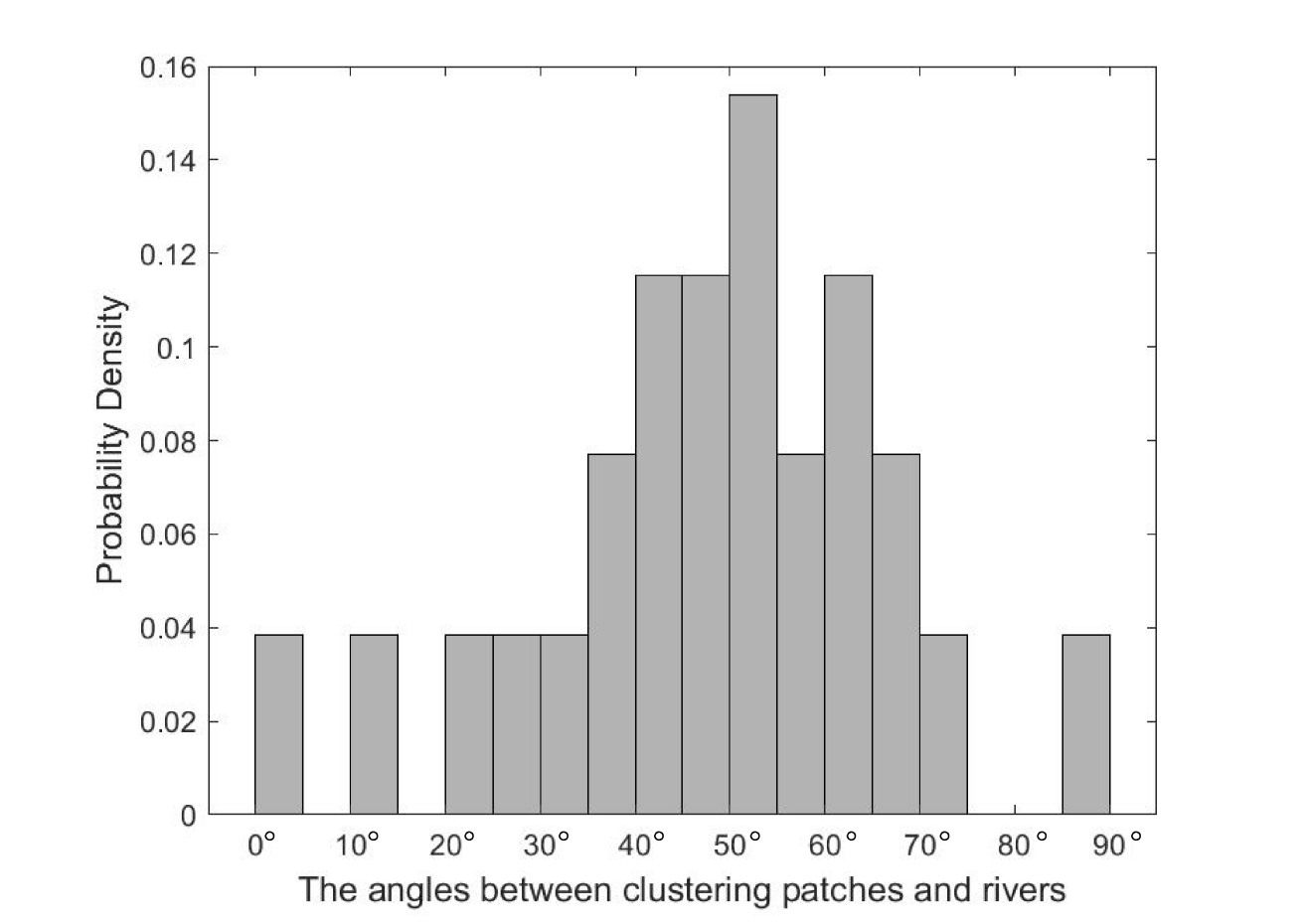


**Figure S3b** Frequency distribution of the angles between the long axis of clustering patches and rivers

**S4 – Revised wind dispersal model**

To provide empirically grounded estimates of wind‑mediated dispersal under realistic extreme conditions, we parameterized the wind dispersal model using long‑term meteorological station data. We used empirical percentiles (95th, 99th, 99.9th) of annual mean wind speeds to drive the simulations. Additionally, we tested the sensitivity of the model to the turbulence coefficient (*ĸ*) to assess the robustness of our conclusions to parameter uncertainty.

**Methods**

Annual mean wind speed data from 2001 to 2022 were obtained from 71 meteorological stations in the study region. Seed terminal velocity was measured in the laboratory following standard methods. Dispersal was modelled using the mechanistic framework of Nathan et al. (2011). Extreme scenarios were defined using empirical percentiles (95th, 99th, 99.9th) of the station‑averaged annual mean wind speed. Turbulence sensitivity was tested by varying the von Kármán constant *κ* from 0.3 to 0.5.

**Results**

The modelled annual seed dispersal speed under the baseline (mean wind) scenario was 0.0871 km/year (range 0.0674‑0.112 km/year). Under the 99.9th percentile wind speed (5.89 m/s), the mean dispersal speed increased only slightly to 0.0976 km/year (range 0.0758‑0.121 km/year) – still two orders of magnitude lower than vehicle‑ or animal‑mediated dispersal (Table S4a). Varying the turbulence coefficient *κ* from 0.3 to 0.5 changed the dispersal speed from 0.0935 to 0.1017 km/year, a change of less than 9% (Table S4b). These results demonstrate that wind dispersal is negligible and that the model is insensitive to turbulence parameters. Figure S4a shows the boxplot of dispersal speeds for different values of the turbulence coefficient *ĸ*.

**Table S4a** Modelled annual seed dispersal speed under empirical wind percentiles

| Wind scenario | Mean dispersal speed (km/year) | Minimum (km/year) | Maximum (km/year) |
| --- | --- | --- | --- |
| Baseline (mean wind) | 0.0871 | 0.0674 | 0.112 |
| 95th percentile | 0.0958 | 0.0751 | 0.119 |
| 99th percentile | 0.0973 | 0.0757 | 0.121 |
| 99.9th percentile | 0.0976 | 0.0758 | 0.121 |

Note: The 99.9th percentile wind speed was 5.89 m/s.

**Table S4b** Sensitivity of dispersal speed to turbulence coefficient *ĸ* (using 99.9th percentile wind speed)

| *ĸ* | Mean dispersal speed (km/year) |
| --- | --- |
| 0.3 | 0.0935 |
| 0.4 | 0.0976 |
| 0.5 | 0.1017 |


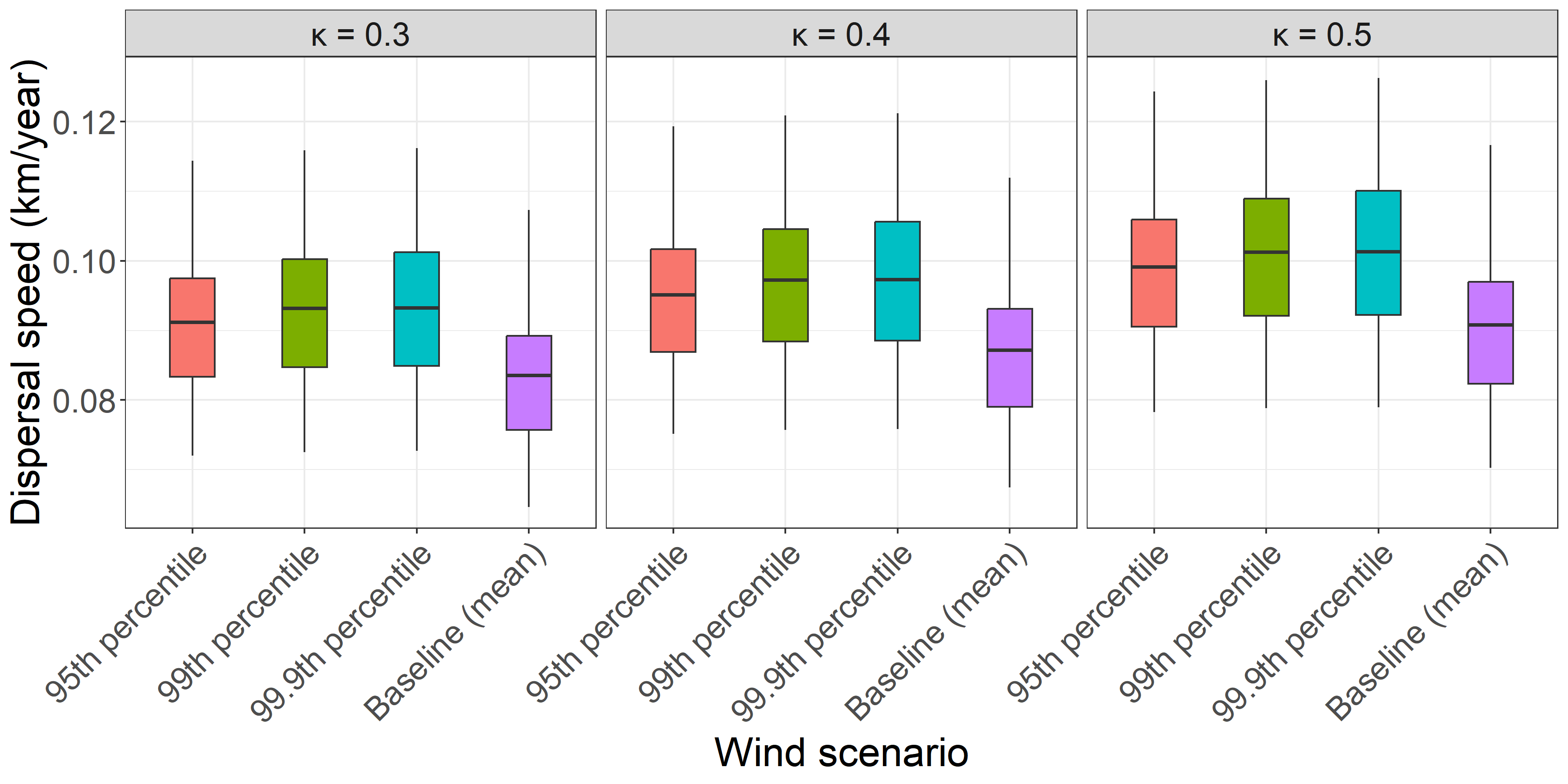


**Figure S4a** Boxplots of wind dispersal speeds across different values of the turbulence coefficient *ĸ* (0.3, 0.4, and 0.5)

**S5 – Vehicle‑mediated seed adhesion and retention dynamics**

To provide a detailed visualisation of vehicle-mediated dispersal potential, we present the full experimental results on seed adhesion probabilities across soil texture classes and distance-dependent retention dynamics, along with the performance of candidate dispersal kernels.

**Methods**

For each of the nine USDA soil texture classes, a slurry was prepared with particle‑size fractions matching natural soils (sand: 50-2000 µm, silt: 2-50 µm, clay: <2 µm). One hundred inactivated *S. rostratum* seeds were scattered on the slurry surface, and a medium-duty flatbed truck (70 km/h) was driven over the soil. Seeds remaining on the vehicle (wheels and mudguards) were collected. Adhesion probability was calculated as (initial seeds − recovered seeds) / initial seeds × 100%. Five replicates were performed per soil type.

Adhered seeds were subjected to driving distances of 0-4096 m (14 intervals). At each distance, dislodged seeds were counted. Retention ratio was defined as (seeds still attached / initially adhered seeds) × 100%.

Four functions were fitted to the retention data: simple exponential, double exponential decreasing, double exponential increasing, and power exponential (Eqs. 4-7 in main text). Model selection was based on R^2^ and bootstrap *P*-values (1000 iterations), implemented in Python.

**Results**

Adhesion probabilities varied by soil texture, ranging from 0.07 (sandy loam) to 0.30 (clay) (Figure S5a). Clay-rich soils retained seeds most effectively, likely due to higher surface tackiness. Retention declined rapidly with distance: at 60 m, average retention was ≈45%; at 256 m, ≈10%; and beyond 1024 m, <2% (Figure S5b). Among the four candidate models, the simple exponential function (Eq. 4) provided the best fit (R^2^= 0.94, *P*< 0.001; Figure S5c), outperforming the double exponential decreasing (R^2^= 0.89; Figure S5d), double exponential increasing (R^2^=0.78; Figure S5e), and power exponential (R^2^=0.85; Figure S5f). The simple exponential model was therefore used to estimate dispersal distances and speeds reported in the main text.


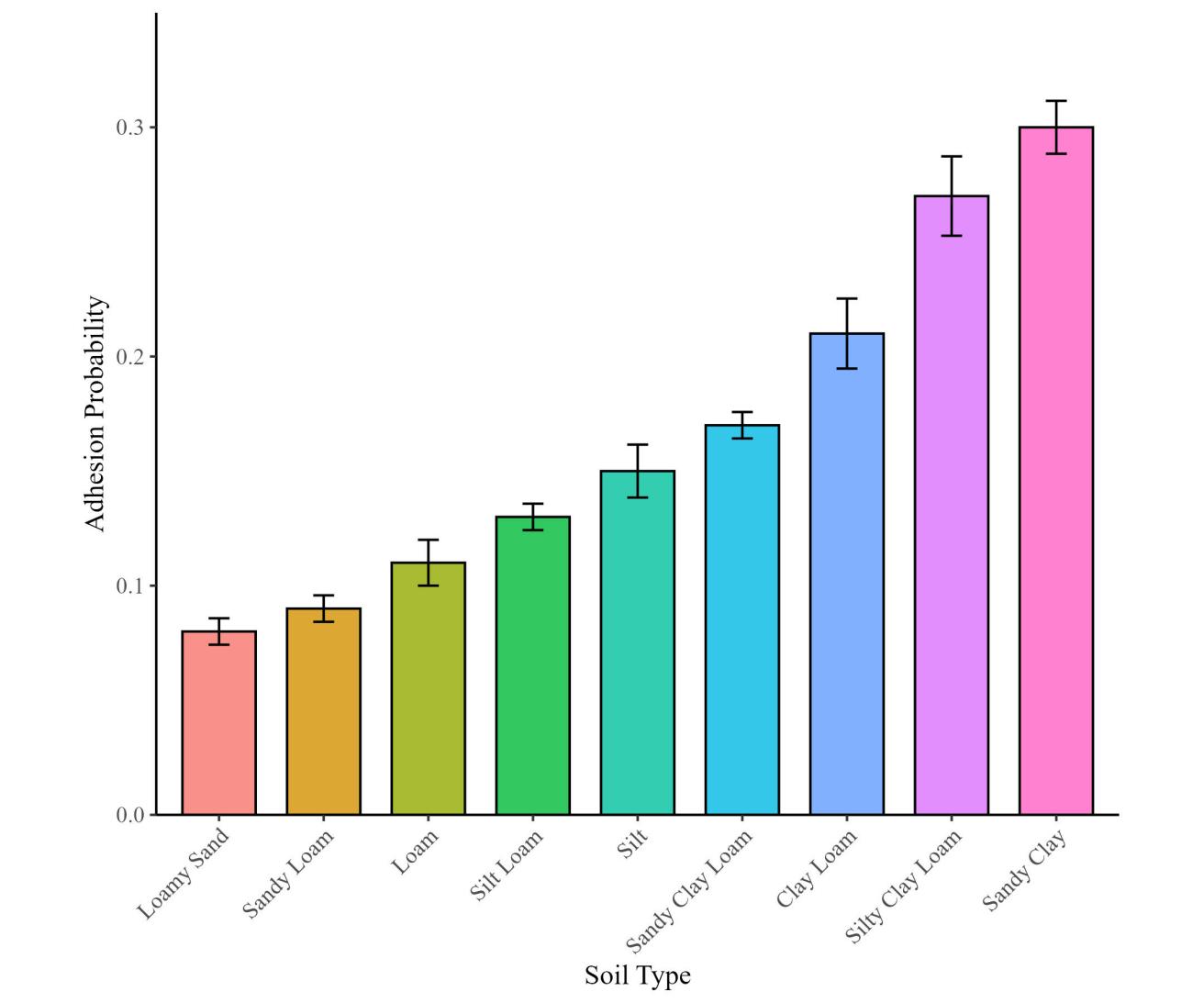


**Figure S5a** Seed-to-vehicle adhesion probabilities across soil texture classes in experimental replicates


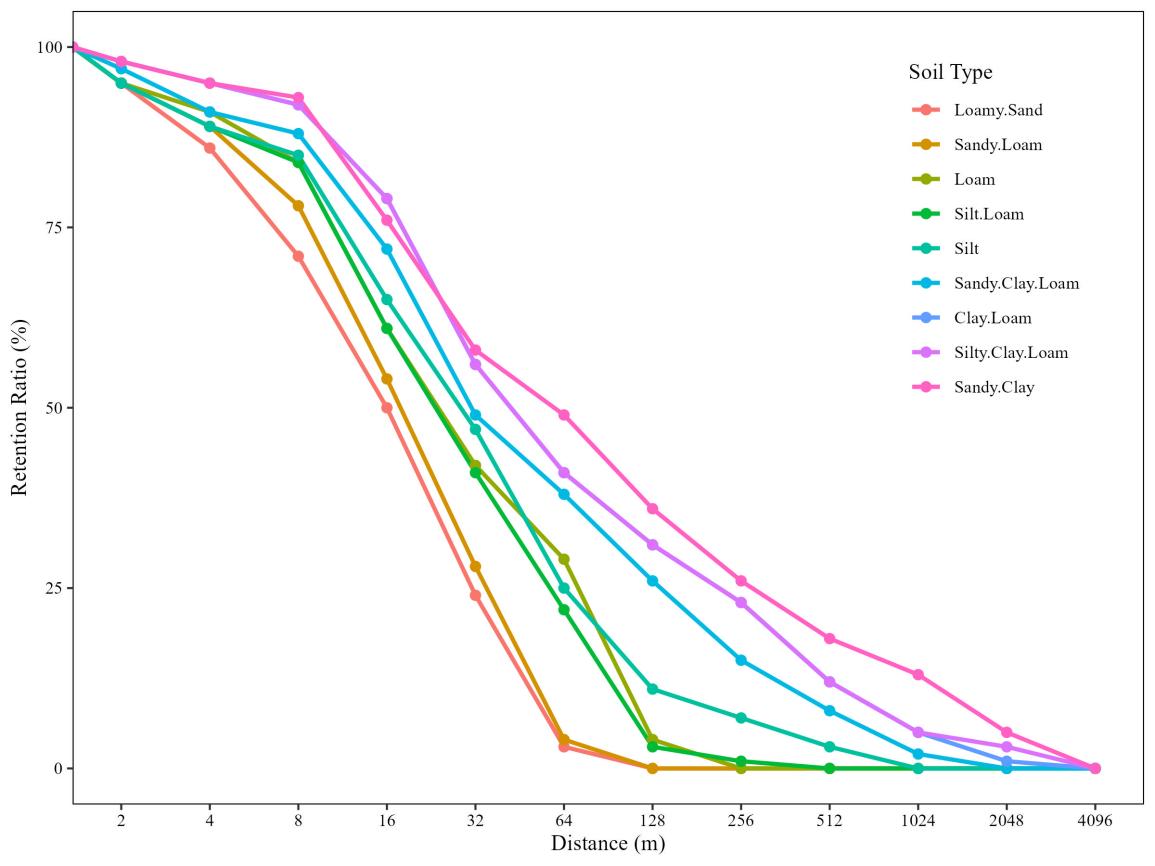


**Figure S5b** Seed retention rates (%) on vehicle surfaces at varying transport distances across nine soil texture classes


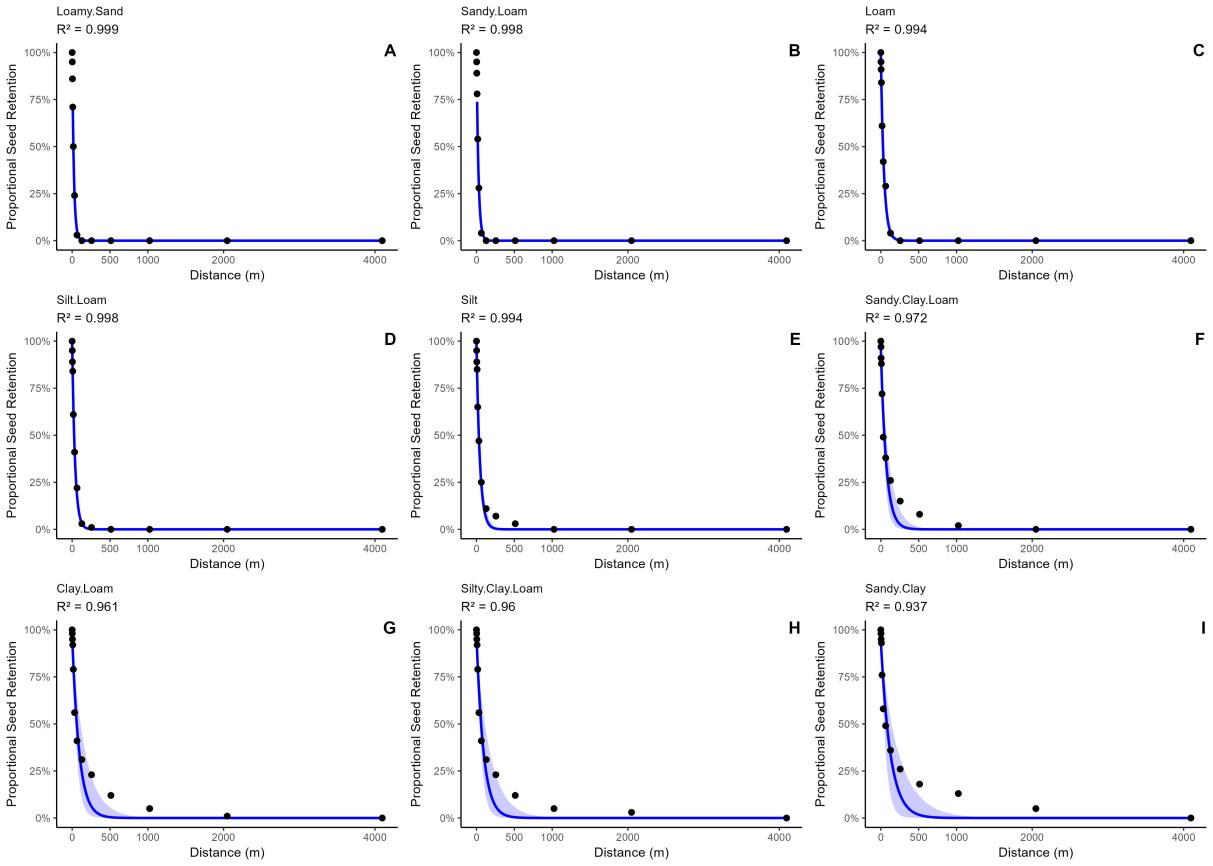


**Figure S5c** Simulation performance of the simple exponential model (Eq. 4) for seed drop rate with vehicle driving distance from the parent plant


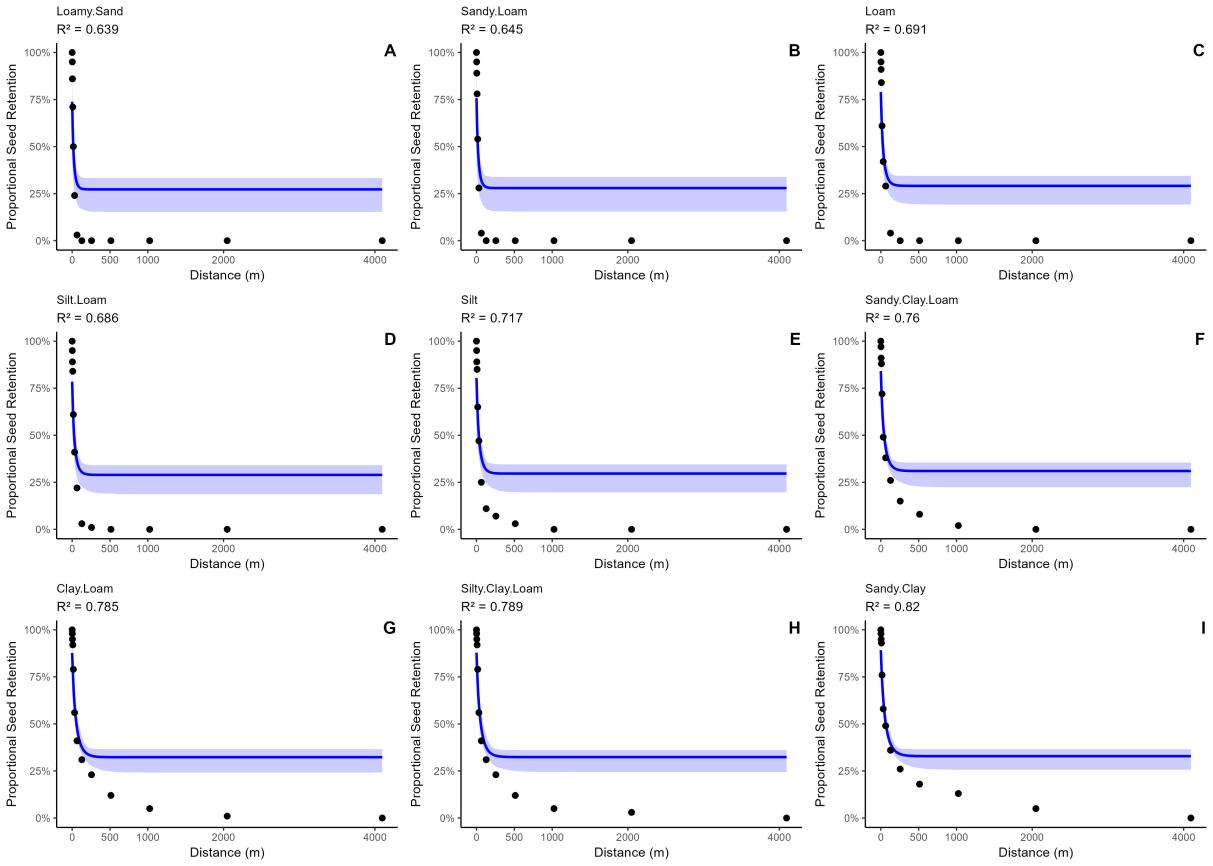


**Figure S5d** Simulation performance of the double exponential model (Eq. 5) for seed drop rate with vehicle driving distance from the parent plant


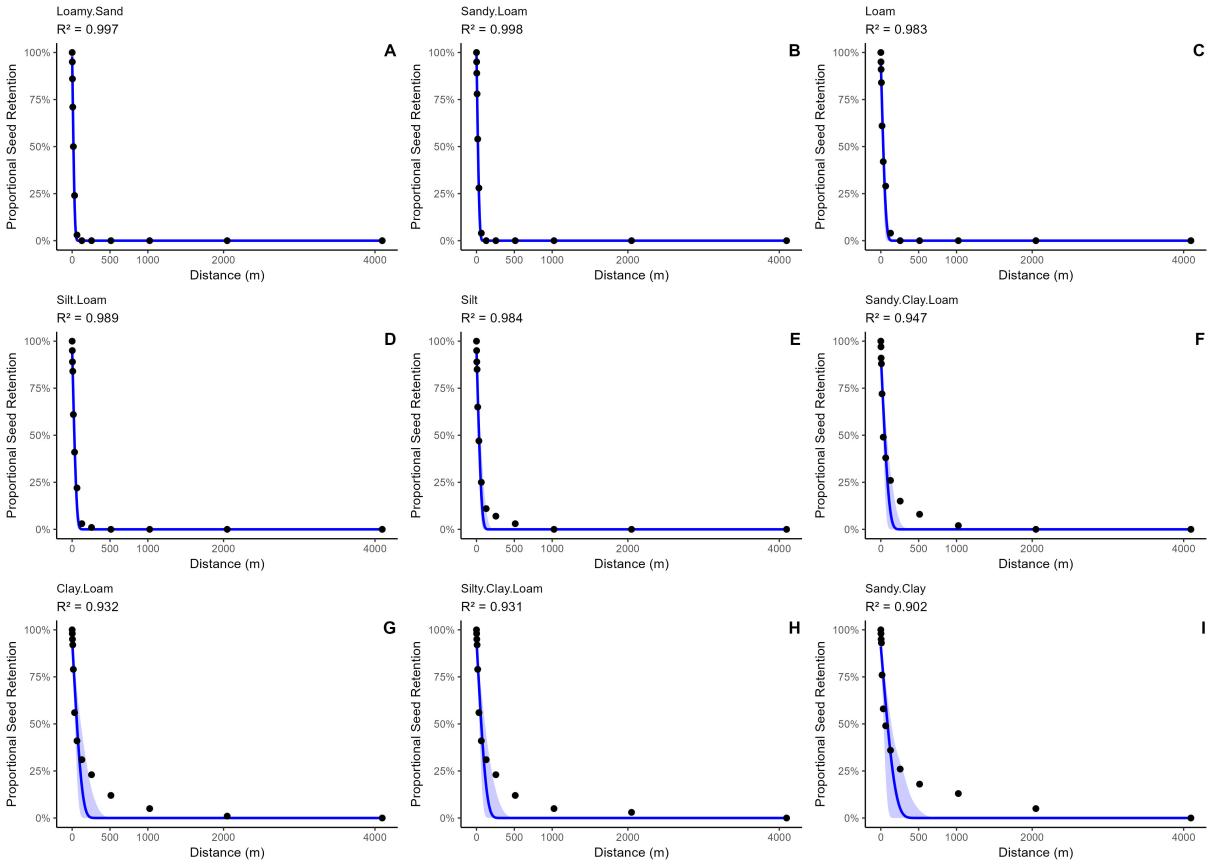


**Figure S5e** Simulation performance of the double exponential model (Eq. 6) for seed drop rate with vehicle driving distance from the parent plant


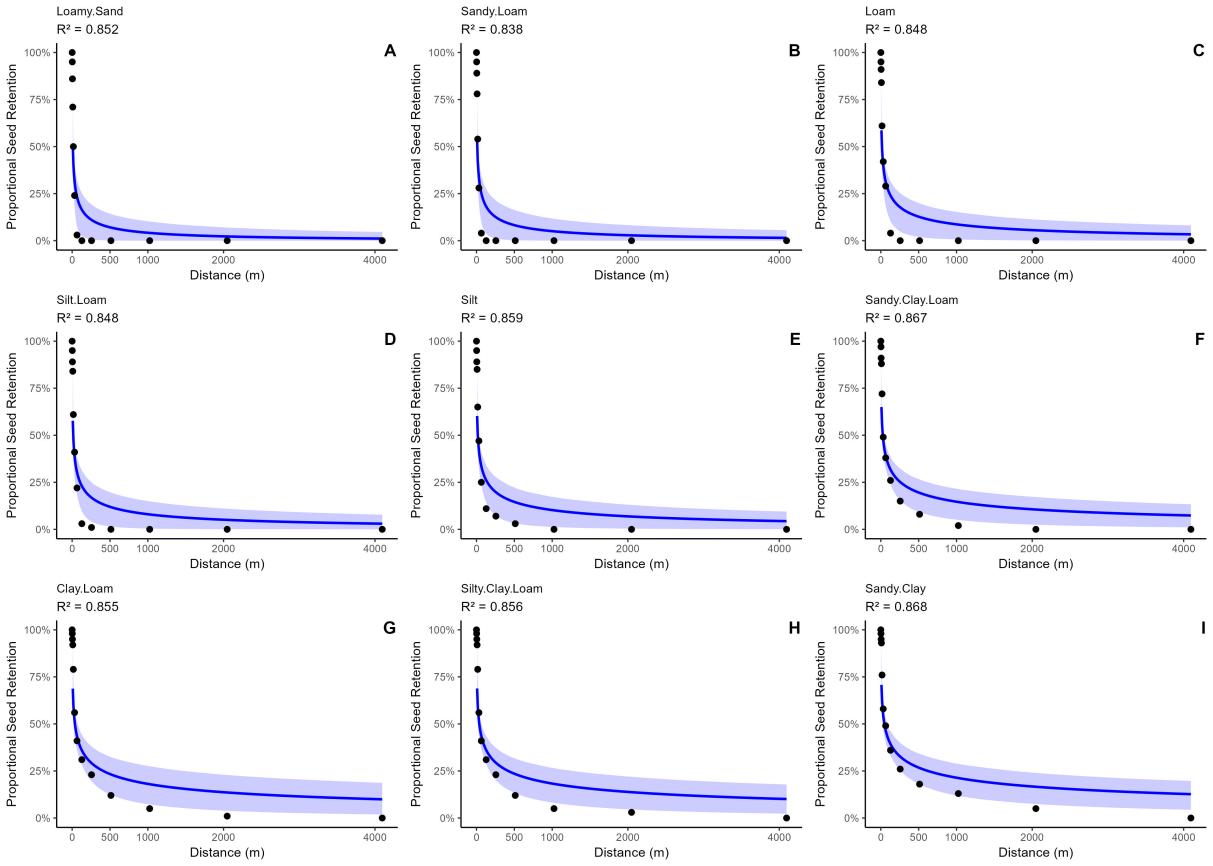


**Figure S5f** Simulation performance of the power exponential function (Eq. 7) for seed drop rate with vehicle driving distance from the parent plant

**S6 – Animal‑mediated epizoochory: fruit retention on sheep wool**

To provide detailed experimental data on distance-dependent fruit retention on sheep wool and model performance, we present the full retention dynamics and comparative fitting of four decay functions.

**Methods**

One hundred fresh S. rostratum fruits were attached to the wool of domesticated sheep (Ovis aries) in the field. Sheep were allowed to graze normally, and the number of fruits remaining attached was recorded at distances of 0.6, 1.2, 2.4, 3.6, and 4.8 km (simulating an 8-hour grazing period). Three replicates were performed. Retention ratio was calculated as fruits remaining / initial fruits × 100%.

Four mathematical functions (simple exponential, double exponential decreasing, double exponential increasing, power exponential) were fitted to the retention data. Model performance was compared using R^2^ and AIC.

**Results**

Fruit retention declined steeply with distance: 92% retention at 0.6 km, 45% at 2.4 km, and only 2% at 4.8 km (Figure S6a). The double exponential decreasing function provided the best fit (R^2^= 0.955), followed by the power exponential (R^2^= 0.93), while the simple exponential performed poorly (R^2^ = 0.72) (Figure S6b). This indicates a biphasic decay pattern, with rapid initial loss followed by slower attrition. The best-fit model was used to estimate effective dispersal distances and speeds in the main text.


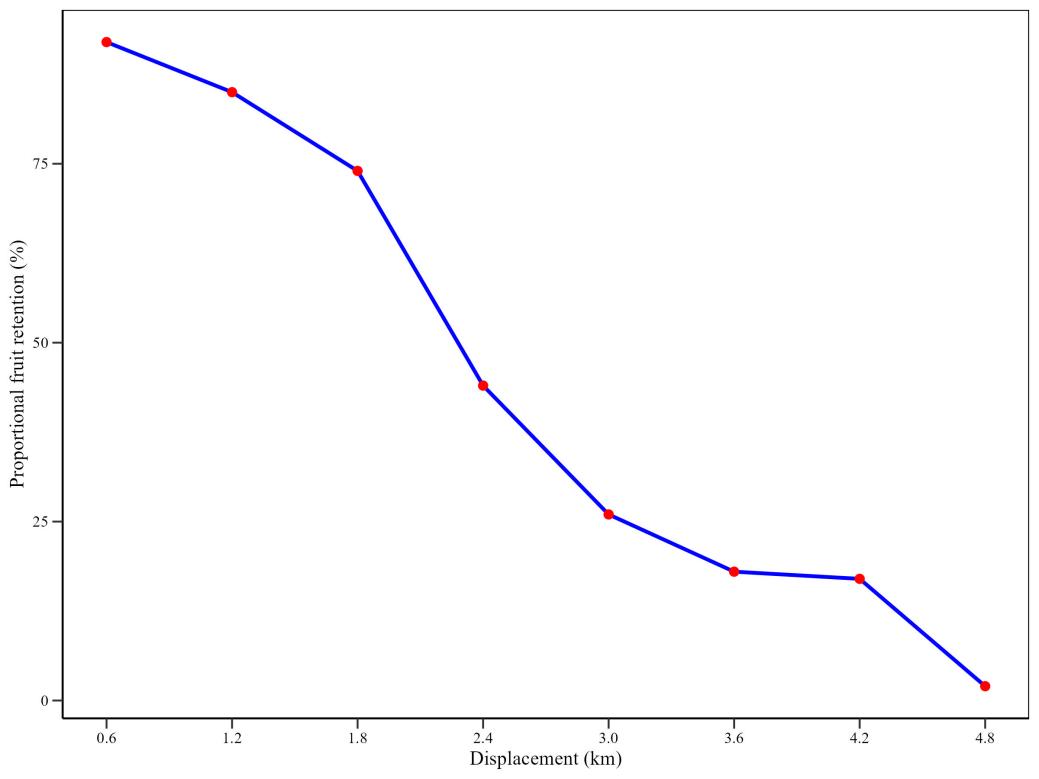


**Figure S6a** Distance-dependent retention of *S. rostratum* fruits on sheep wool


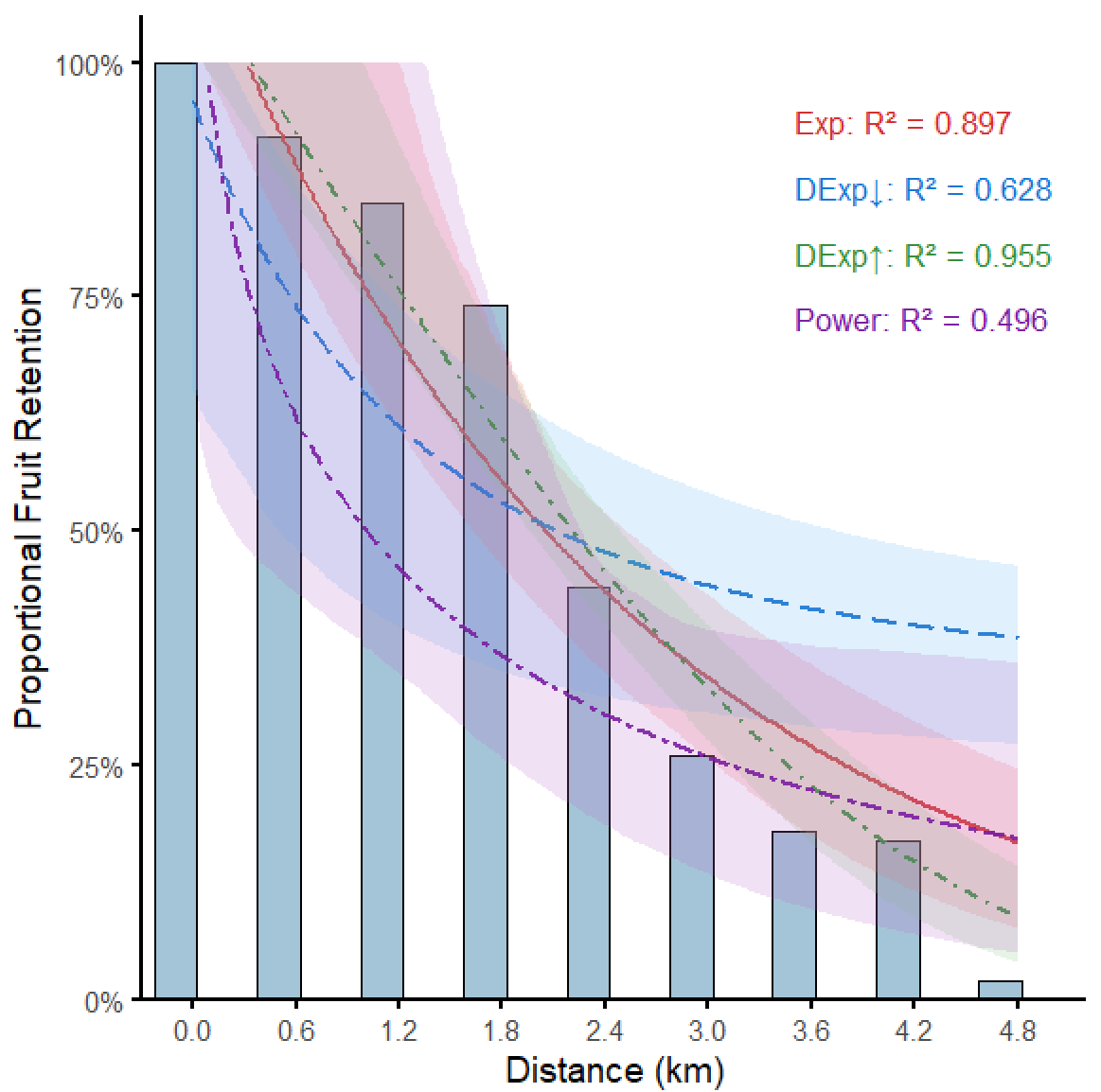


**Figure S6b** Differential performance of four mathematical decay functions in characterizing seed retention dynamics

**S7 – Power analysis for endozoochory (frequentist)**

To address the statistical limitation of inferring complete absence of endozoochory from a small sample (six sheep, zero germination), we performed a post‑hoc power analysis to determine the minimum true seed survival rate detectable with 95% confidence.

**Methods**

A post‑hoc power analysis was performed for a binomial model. Given *n*=6 sheep and an observed outcome of zero germinations (*x*=0), the minimum true survival rate *p*_min_ that would have been detected with 95% confidence (i.e., probability of observing zero germinations <0.05) is calculated as:

$$p_{min}=1-\alpha^{1/n}, with \alpha=0.05$$

**Result**

The calculation yields $p_{min}=1-{0.05}^{1/6}\approx0.393$. This means the experiment can only rule out true seed survival rates greater than 39.3%. Lower survival rates (e.g., 5%, 10%) cannot be statistically excluded. Therefore, while the trial suggests a strong lethal effect of sheep digestion, a low level of endozoochory remains possible.

**S8 – Distribution of minimum arrival speed (MAS) within and between patches**

To select an appropriate statistical model for analysing the drivers of spread speed, we first conducted an exploratory data analysis (EDA) of the empirical distribution of minimum arrival speeds (MAS) calculated from occurrence records. This analysis characterizes the shape, skewness, and range of the response variable, which informs the choice of error distribution and link function in subsequent regression modelling.

**Methods**

The distribution of MAS values (intra- and inter-patch combined) was visualised using a histogram with a kernel density overlay. A log-transformation was applied to assess whether normality could be achieved. Normality was tested using the Shapiro-Wilk test. All visualisations and tests were performed in R (version 4.2.1) using ggplot2 and stats packages.

**Results**

The raw MAS values exhibited a strongly right-skewed distribution, with a long tail extending to high speeds (Figure S8a). The boxplot (Figure S8a) shows a median near 0.8 km/year and numerous outliers beyond 10 km/year, confirming the non-normal nature of the data. Log-transformation (Figure S8c) improved symmetry but did not fully achieve normality (Shapiro-Wilk test, *P* < 2.2e-16). These results support the use of a Gamma GLMM with a log-link function, which is appropriate for positive, continuous, right-skewed data without requiring transformation of the response variable.


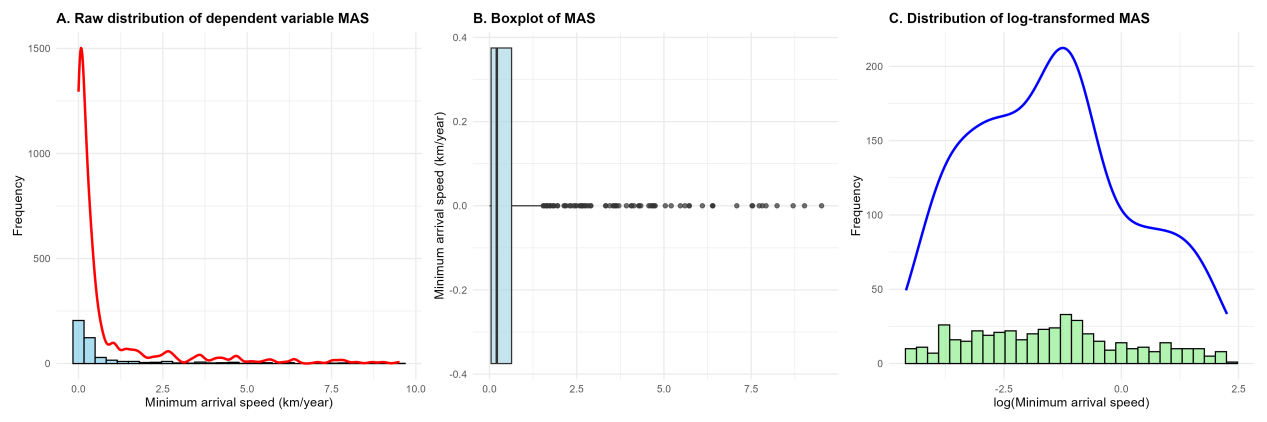


**Figure S8** Distribution of minimum arrival speed (MAS). (A) Histogram and density plot of raw MAS, showing pronounced right skew. (B) Boxplot of raw MAS, displaying median and outliers. (C) Histogram and density plot of log-transformed MAS.

**S9 – LMG variance decomposition and threshold sensitivity analysis**

To interpret the relative importance of the three dispersal vectors in the Gamma GLMM, we applied a validated method for decomposing explained variance. Additionally, because the patch classification thresholds in the main text (dominance: 60%; major: 40%) are inherently arbitrary, we assessed the stability of patch assignments to different dispersal mechanism categories across a range of threshold values.

**S9.1 LMG variance decomposition**

**Methods**

The LMG (Lindeman-Merenda-Gold) method was applied to the fixed-effect components of the Gamma GLMM (wind, vehicle, and animal epizoochory potentials). The decomposition was performed on linear predictor scale. Marginal R^2^ (fixed effects) was 0.593; conditional R^2^ (fixed + random) was 0.643. The absolute contribution of each predictor was calculated as the proportion of the total variance explained (conditional R^2^) attributed to that predictor when added sequentially, averaged over all orderings.

**Result**

Vehicle dispersal made the largest contribution to the explained variance, with an LMG contribution of 0.239 (55.3% of the relative contribution among the three vectors). Animal-mediated epizoochory contributed 0.187 (43.3%), while wind contributed only 0.0062 (1.43%). These proportions are consistent with the effect sizes and significance levels reported in the main text. The LMG decomposition confirms that vehicle dispersal is the dominant single vector explaining variation in spread speed, followed by animal epizoochory, with wind playing a negligible role.

**S9.2 Threshold sensitivity analysis for patch classification**

**Methods**

The original patch classification used a dominance threshold of 60% and an interaction (major) threshold of 40%. To test whether the classification is sensitive to these choices, we varied the dominance threshold (50%, 55%, 60%, 65%, 70%) and the interaction threshold (35%, 40%, 45%), creating 15 combinations. For each combination, patches were re‑classified, and concordance (proportion of patches assigned to the same category as the original classification) was calculated.

**Results**

The average concordance across all 15 threshold combinations was 79.7%, indicating that the classification is generally robust. When restricting to reasonable variations around the original thresholds (dominance = 55-65%, major = 40-45%), concordance exceeded 85%. No threshold combination produced a wind-only patch, consistent with the conclusion that wind never acts as a sole dominant vector. The original thresholds (dominance = 60%, major = 40%) are therefore representative and do not bias the qualitative conclusions regarding the prevalence of different dispersal mechanisms.

**References**

MacKenzie, D. I., Nichols, J. D., Lachman, G. B., Droege, S., Royle, J. A., & Langtimm, C. A. (2002). Estimating Site Occupancy Rates When Detection Probabilities Are Less Than One. Ecology, 83(8), 2248–2255. https://doi.org/10.2307/3072056

Nathan, R., Horvitz, N., He, Y., Kuparinen, A., Schurr, F.M., Katul, G.G. (2011). Spread of North American wind-dispersed trees in future environments. Ecol. Lett. 14(3), 211–219. https://doi.org/10.1111/j.1461-0248.2010.01573.x.
